# Developmental and Environmental Regulation of *Aquaporin* Gene Expression across *Populus* Species: Divergence or Redundancy?

**DOI:** 10.1371/journal.pone.0055506

**Published:** 2013-02-05

**Authors:** David Cohen, Marie-Béatrice Bogeat-Triboulot, Silvère Vialet-Chabrand, Rémy Merret, Pierre-Emmanuel Courty, Sébastien Moretti, François Bizet, Agnès Guilliot, Irène Hummel

**Affiliations:** 1 INRA, UMR1137 Ecologie et Ecophysiologie Forestières, Champenoux, France; 2 Université de Lorraine, UMR1137 Ecologie et Ecophysiologie Forestières, Faculté des Sciences, Vandœuvre-lès-Nancy, France; 3 Zürich-Basel Plant Science Center, Botanical Institute, University of Basel, Basel, Switzerland; 4 Vital-IT, SIB Swiss Institute of Bioinformatics, Quartier Sorge, bâtiment Génopode, Lausanne, Switzerland; 5 Department of Ecology and Evolution, bâtiment Biophore, Lausanne University, Lausanne, Switzerland; Instituto de Biología Molecular y Celular de Plantas, Spain

## Abstract

Aquaporins (AQPs) are membrane channels belonging to the major intrinsic proteins family and are known for their ability to facilitate water movement. While in *Populus trichocarpa*, AQP proteins form a large family encompassing fifty-five genes, most of the experimental work focused on a few genes or subfamilies. The current work was undertaken to develop a comprehensive picture of the whole *AQP* gene family in *Populus* species by delineating gene expression domain and distinguishing responsiveness to developmental and environmental cues. Since duplication events amplified the poplar *AQP* family, we addressed the question of expression redundancy between gene duplicates. On these purposes, we carried a meta-analysis of all publicly available Affymetrix experiments. Our in-silico strategy controlled for previously identified biases in cross-species transcriptomics, a necessary step for any comparative transcriptomics based on multispecies design chips. Three poplar *AQPs* were not supported by any expression data, even in a large collection of situations (abiotic and biotic constraints, temporal oscillations and mutants). The expression of 11 *AQPs* was never or poorly regulated whatever the wideness of their expression domain and their expression level. Our work highlighted that *PtTIP1;4* was the most responsive gene of the *AQP* family. A high functional divergence between gene duplicates was detected across species and in response to tested cues, except for the root-expressed *PtTIP2;3/PtTIP2;4* pair exhibiting 80% convergent responses. Our meta-analysis assessed key features of aquaporin expression which had remained hidden in single experiments, such as expression wideness, response specificity and genotype and environment interactions. By consolidating expression profiles using independent experimental series, we showed that the large expansion of *AQP* family in poplar was accompanied with a strong divergence of gene expression, even if some cases of functional redundancy could be suspected.

## Introduction

Aquaporins (AQPs) are found in every organism but are especially abundant in plants [1]. In higher plants, AQPs have been classified into five subfamilies: plasma membrane intrinsic proteins (PIPs), tonoplast intrinsic proteins (TIPs), nodulin-26 intrinsic proteins (NIPs), small basic intrinsic proteins (SIPs) and unrecognized X intrinsic proteins (XIPs). These intrinsic channel proteins facilitate and regulate the passive movement of water molecules and other small neutral molecules (e.g. urea, glycerol, ammonium, metalloids) across biological membranes [2], [3]. AQPs are involved in major physiological processes such as root and leaf hydraulic plasticity, stomatal aperture, cell expansion, or acclimation to drought or salinity [3]. Some isoforms play important roles in other processes such as gas or nutrient uptake and translocation, and nitrogen remobilisation [4], [5]. The increase of AQP isoforms in plants has been suggested to “offer adaptive advantages for growth in different environmental conditions, possibly as a result of divergent transport selectivities or regulatory mechanisms” [6]. Although regulation of AQP activities relies on a complex interplay of post-transcriptional, translational and post-translational processes [7], monitoring gene expression has been a valuable tool to dissect AQP roles in plant functioning [8]–[10].

Fifty-five *AQP* genes were identified in *Populus trichocarpa* genome [11]. One of the main rationale motivating analyses of gene expression in *Populus* comes from its status of model system characterised by woodiness and perennial habit and thus developing structures and behaviours which are not questionable in herbaceous and annual models [12], [13]. In addition, *P. trichocarpa* has become a model to study the evolution of duplicated genes, the Salicoid duplication event having significantly contributed to the amplification of multigene families [14]–[16]. In various *Populus* species, regulations of *AQP* expression were reported during adventitious root development in *P. trichocarpa x deltoides* [17], mycorrhizal symbiosis in *P. tremula x tremuloides* [18] and recovery from xylem embolism in *P. trichocarpa* [19]. Some *AQP* members were found responsive to environmental challenges and hormone treatments (in *P. balsamifera, P. simonii x balsamifera, P. alba x tremula, P. trichocarpa x deltoides*) [20]–[23] and to be differentially expressed depending on genotypes [24]. Most of these studies focused on a few *AQP* genes and/or subfamilies. In several analyses of whole transcriptome response, some *AQP* members were listed among the most responsive genes to various environmental constraints [25]–[28]. Meanwhile, the sole family-wide picture of *AQP* expression drawn to date has been a visualization of transcript accumulation across nine tissues from *Populus balsamifera* [11].

Our aim was to provide new insights for functional characterisation of the *AQP* gene family in *Populus* by delineating their expression domain and distinguishing their responsiveness to developmental and environmental cues. Taking advantage from the large expression data set obtained with the Affymetrix GeneChip Poplar Genome Array, several sources of diversity were simultaneously investigated, namely species/genotypes, tissues/organs and various cues. *In-silico* strategy was optimised to control for previously identified biases in cross-species transcriptomics [25], [29]. Key aspects of *AQP* expression profiles were cross-validated using previously-published data such as expressed sequence tag libraries (EST), expression data from qPCR or from another platform array (GPL7424, NCBI, Gene Expression Omnibus). Our meta-analysis reveals the specificities of *AQP* expression which cannot be fully addressed in single experiments, such as expression wideness, response specificity as well as genotype-dependent diversity. Through the simultaneous investigation of experimental series, we show that the large expansion of *AQP* family in poplar was accompanied with a strong divergence of gene expression, even if some cases of functional redundancy could be suspected.

## Materials and Methods

### Database Search

Full-length sequences of all *AQP* genes of *Populus trichocarpa* were downloaded from Phytozome v8.0 [30]. A total of 429,444 *Populus* expressed sequence tags (EST) were downloaded from the GenBank database [31]. *AQP* coding regions were used as queries to perform BLASTN alignment against all EST [32]. NCBI BLAST 2.2.25+ executable was used on a local platform. Command line “blastn” was executed with task argument set as “blastn” and default parameters (word size: 11, expect threshold: 10, match/mismatch scores: 2/−3, gap penalties: existence 5, extension 2). Matches above 96% identity and over an alignment of at least 100 bp were considered as corresponding sequences of *AQPs*. Reverse BLASTN strategy (using EST as queries against *AQP* transcripts) was performed to assign each EST to a single *AQP* ID. Metadata associated to each EST were manually inspected for their tissue origin.

All publicly available Affymetrix GeneChip Poplar Genome Array data were downloaded from the NCBI Gene Expression Omnibus [33] and ArrayExpress [34] at the end of January 2012. Collection gathered 632 arrays from distinct experiments. Within each experiment, arrays were normalised with the GcRMA package (GcRMA 2.0 [35]) available in Bioconductor [36], followed by Log2 transformation and calculation of the mean for each condition [16]. *AQP* expression was explored in a subset of 110 “control” arrays, excluding “treatment” and “transgenic line” data. We discriminated eight sample types, namely suspension cells, seedling, catkin, shoot apex, leaf, stem, root and xylem. To analyse regulation of *AQP* expression, mean signal intensities were pair-wise compared and expressed as Log2 ratio. To prevent introduction of noise, computation of Log2 ratio was constrained, i.e. set to null when signal intensities of the two compared conditions were below background level (cut-off set to 3.2). Treated plants or “transgenic lines” were compared to their respective control or wild type. In the analysis of temporal series, successive time points were compared to the initial one (ie t = 0 or predawn). The present meta-analysis comprises 167 comparisons.

The Affymetrix GeneChip Poplar Genome Array contains 61,251 probe sets representing over 56,000 transcripts and predicted genes, and was generated from several *Populus* species (including *P. trichocarpa* genome v1.1). Probe sets corresponding to *AQPs* were identified using Batch Query and Probe Match, tools available at the NetAffx Analysis Center (http://www.affymetrix.com). “Batch Query” was run either using Gene Symbol from previous releases of *P. trichocarpa* genome (v1.1 and v2.0) or NCBI RefSeq. “Probe Match” found probes that identically match *AQP* sequences. Due to the criterion used for the array design (minimal overlap between EST/mRNA-based UniGene clusters and predicted genes), some probe sets were lacking of a gene model correspondence. To strengthen our annotation, the 61,251 target sequences (i.e. one per probeset on the array) were confronted to *P. trichocarpa* genome. Target sequences of probe sets were used as queries against *P. trichocarpa* genome, using a local BLASTN with parameters mentioned above. Each probeset were re-annotated according to the best BLAST hits per query.

### Extracting Gene-level Information from Probe Set-based Information

Extracting reliable gene-level information from probe set-based information is still under debate [25]. While the use of median value takes advantage from the presence of multi-probe sets for automatic consolidation, it relies on the assumption that distinct probe sets are equally suitable for the detection of a given transcript (at least having equivalent matching probabilities). This assumption stands as long as probe sets x sample matrices are homogeneous [37]. Our analysis being based on experiments carried on distinct poplar species hybridised on a multispecies-designed array, one could expect that probe sets designed on EST from different species would differentially match depending on species matrices. We tested this hypothesis by screening signal intensity and Log2 ratio of all probe sets targeting a given gene and comparing information retrieved from median and maximal values (Figure S1). The two methods were mostly consistent. Median provided lower estimates of expression and/or regulation than maximum since it took into account absence of signal. Median depended not only on the number of probe set per gene but also on the compatibility between probe set and hybridised matrix, which makes it unsuitable in a meta-analysis (illustrated for *PtPIP2;4 -* Figure S1). Expression and regulation for each gene were thus extracted from probe set data using maximal values (either maximal signal intensity or maximal absolute Log2 ratio). Data were visualised by heatmap and hierarchical clustering, which was performed with 'hclust' function using Euclidean distance (R2.14.1, http://www.R-project.org). Based on the Log2 ratio distribution, regulations of gene expression were categorized according to their intensity applying a fold change threshold of 1.5 (fold change = 2^Log2ratio^).

### Sequence Analysis

Phylogenetic relationships of AQP family have been previously described [11]. *Populus* genome had undergone several rounds of genome-wide duplication followed by multiple segmental and tandem duplications [13], [15]. Among them, the Salicoid duplication event had significantly contributed to the amplification of multigene families. Three interfaces were interrogated to identify duplicate pairs in the poplar *AQP* family (Gramene release 34b, http://gramene.org; PGDD [38]; Plaza v2.5 [39]). The genetic distance between syntenic gene pairs was examined on the basis of the proportion of four-fold degenerate nucleotide sites that underwent transversions (4DTV values) [13]. The 4DTV values were downloaded from Plaza v2.5 and from a recent genome-wide analysis of gene pair in poplar [15]. Synonymous (dS) and nonsynonymous (dN) substitution rates were estimated from nucleotide sequences in a pair-wise manner with CodonSuite interface [40].

## Results and Discussion

### The Aquaporin Family in *Populus trichocarpa* Genome

Gupta and Sankararamakrishnan [11] studied the *AQP* family in the *Populus trichocarpa* genome v1.1. They discarded nine invalid sequences, confirmed 54 *AQP* genes and identified a new *AQP* sequence. In subsequent versions of *P. trichocarpa* genome (v2.0 and v2.2), the functional annotation “Aquaporin” has been consistently up-dated, except two remaining invalid sequences (*POPTR_0007s07950* and *POPTR_1606s00200* [11]). *PtXIP1;1* has been recently invalidated (truncated sequence *POPTR_0009s13100*) [23]. We thus considered 54 predicted genes and used the nomenclature of Gupta and Sankararamakrishnan [11]. Using these genomic sequences, we detected 2961 expressed sequence tags (EST).

Closely related *AQP* pairs were identified in previous phylogenetic analysis [11], [24]. The expansion of the *AQP* family in *P. trichocarpa* genome resulted from both segmental and tandem duplications (Table 1). Only six *AQP* genes could be considered as single copy in *P. trichocarpa* genome (*PtNIP1;3* - *PtNIP1;4* - *PtNIP1;5* - *PtNIP3;5* - *PtPIP1;3 - PtXIP2;1*). A lack of congruency across distinct information sources were detected for two clusters (*PtSIP1;3*/*PtSIP1;4* - *PtTIP3;1/PtTIP3;2*). Two *AQP* pairs were retained following tandem duplication processes (*PtPIP2;9/PtPIP2;10* - *PtPIP2;5/PtPIP2;6*). The peculiar mapping of *XIP1s* among *P. trichocarpa* linkage groups (*PtXIP1;3* is located on LGIV while the three others genes are arranged head-to-tail on LGIX), precluded inferring the evolutionary processes that shaped the *PtXIP1* subfamily. The genetic distance between pairs, that was determined on the basis of 4DTV values [13], indicated that several rounds of segmental duplication have shaped the poplar *AQP* family. *PtNIP2;1*, *PtPIP2;8* and *PtTIP4;1* shared complex evolutionary relationships with other members of their subfamily, indicating ancient duplication events. The Salicoid whole-genome duplication event strongly amplified the *AQP* family (16 pairs, Table 1). To explore *AQPs* divergence, the rates of non-synonymous (dN) and synonymous (dS) nucleotide substitutions were calculated (Table 1). dN/dS ratios ranked from 0.05 (*PtTIP1;3/PtTIP1;4*) to 0.47 (*PtSIP2;1/PtSIP2;2*), and were close to those observed within the poplar *HD-ZIP* family [16]. While the highest dN/dS ratios indicated that some pairs may have evolved more rapidly than others following duplication events, a limited functional divergence occurred between AQP pairs, at least in the coding region.

**Table 1:** Non-synonymous/synonymous ratio for AQP pairs.

Gene pairs	duplication	dN	dS	dN/dS
*PtNIP1;1/PtNIP1;2*	S	0.062	0.293	0.212
*PtNIP3;1/PtNIP3;2*	S	0.031	0.271	0.115
*PtNIP3;3/PtNIP3;4*	S	0.042	0.246	0.172
*PtPIP1;1/PtPIP1;2*	S	0.027	0.273	0.098
*PtPIP1;4/PtPIP1;5*	S	0.101	0.280	0.362
*PtPIP2;1/PtPIP2;2*	S	0.053	0.297	0.177
*PtPIP2;3/PtPIP2;4*	S	0.056	0.294	0.191
*PtPIP2;5/PtPIP2;7*	S	0.036	0.368	0.098
*PtPIP2;5/PtPIP2;6*	T	0.008	0.022	0.347
*PtPIP2;9/PtPIP2;10*	T	0.166	0.430	0.386
*PtSIP1;1/PtSIP1;2*	S	0.095	0.223	0.423
*PtSIP1;3/PtSIP1;4*	Nd	0.044	0.202	0.217
*PtSIP2;1/PtSIP2;2*	S	0.087	0.186	0.466
*PtTIP1;1/PtTIP1;2*	S	0.037	0.369	0.099
*PtTIP1;3/PtTIP1;4*	S	0.016	0.325	0.050
*PtTIP1;5/PtTIP1;6*	S	0.031	0.345	0.090
*PtTIP1;7/PtTIP1;8*	S	0.059	0.288	0.203
*PtTIP2;1/PtTIP2;2*	S	0.032	0.217	0.150
*PtTIP2;3/PtTIP2;4*	S	0.029	0.310	0.093
*PtTIP3;1/PtTIP3;2*	Nd	0.046	0.288	0.160
*PtTIP5;1/PtTIP5;2.*	S	0.033	0.211	0.157
*PtXIP1;3/PtXIP1;4*	Nd	0.110	0.531	0.208
*PtXIP1;3/PtXIP1;5*	Nd	0.242	1.240	0.195
*PtXIP1;1/PtXIP1;2* *	T	0.017	0.021	0.824

Gene pairs resulted from segmental (S) or tandem (T) duplication. For two *AQP* pairs no unambiguous inference about duplication events can be provided (Nd).

* *PtXIP1;1* is a pseudogen.

### Extracting *AQP* Information from Affymetrix Data

**Figure 1:**
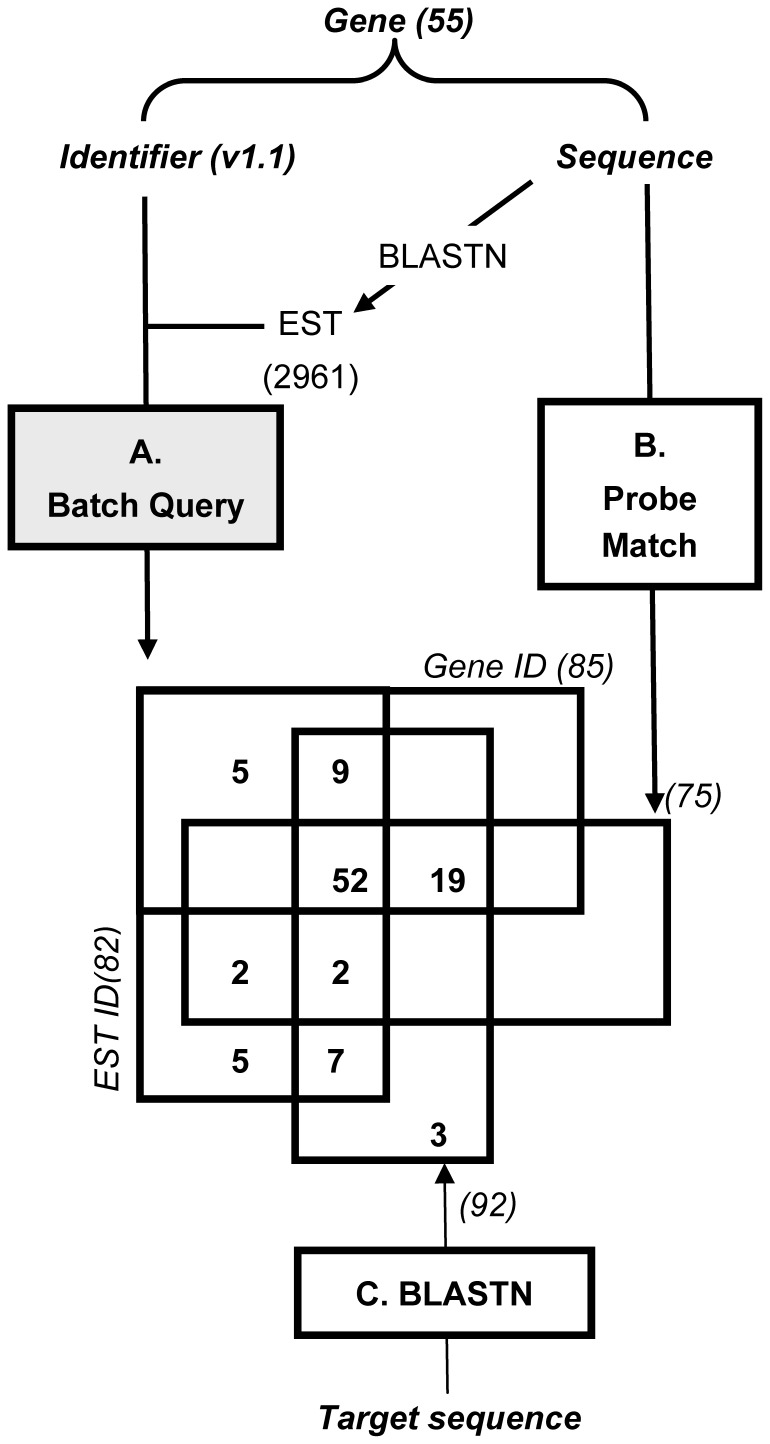
Bio-informatics strategy for GeneChip screening. AQP-targeting probe sets were identified using “Batch Query” and “Probe Match”, tools available as at the NetAffx Analysis Center. **A**. “Batch Query” was run either using Gene symbol or JGI transcript ID and NCBI RefSeq. **B.** “Probe Match” found probes that identically match AQP-coding sequences. **C**. AQP-targeting probe sets were identified through BLASTN alignment of target sequence and Populus trichocarpa genome sequences (v1.1 and v2.0). Venn diagram exhibits the number of probe sets retrieved from each procedure.

*AQP*-targeting probe sets were retrieved on the Affymetrix GeneChip Poplar Genome Array using several identifiers in a three-step strategy (Figure 1). Six probe sets matching invalid *AQP* sequences were filtered out (Table S1). As shown in Figure 1, 85 and 82 probe sets were identified using Gene ID or EST ID, respectively. This discrepancy reflects the multispecies design of the array and the incomplete gene prediction on genomic sequence (such as the lack of UTR prediction [11]). Running Probe Match with *P. trichocarpa AQP* sequences enabled the detection of 75 probe sets. Probe Match revealed only perfect identity – implying that hybridised sequence is known and/or was used in array design. To consider high similarity rather than identity, the 61,251 Affymetrix target sequences were confronted to *P. trichocarpa* genome (v1.1 and v2.0) using BLASTN. We confirmed 89 previously detected probe sets and revealed three new ones. As a final step, the *AQP*-targeting probe sets were *in-silico* evaluated. Three out of seven probe sets commonly detected by EST ID and BLASTN, and one out of the five probe sets found exclusively with EST ID were designed on minus strand and were discarded (Table S1). We also filtered out five probe sets for which gene assignation was ambiguous and one designed in an intronic region. Finally, 94 probe sets were deemed appropriate for targeting 53 *AQPs*, only *PtNIP1;5* being missed. In details, these probe sets were designed on 12 *Populus* species, with a complex layout of species and redundancy as one-to-one probe set to gene relationships concerned only 31 *AQPs* (Table S2). Our analysis highlighted that retrieving information about a multigene family, or even a single gene, on multispecies-designed array cannot only rely on ID Query or Perfect Match but requires similarity-based screening (Figure 1). After proper filtering, the Affymetrix GeneChip Poplar Genome Array appeared to be a valuable tool for *AQPs* profiling. Besides, our optimised *in-silico* strategy is applicable to any multigene family and is suitable for any comparative transcriptomics based on multispecies-designed chips (such as most plant Affymetrix GeneChips).

### Profiling Reveals Tissue- or Organ-preferred Expression

**Figure 2:**
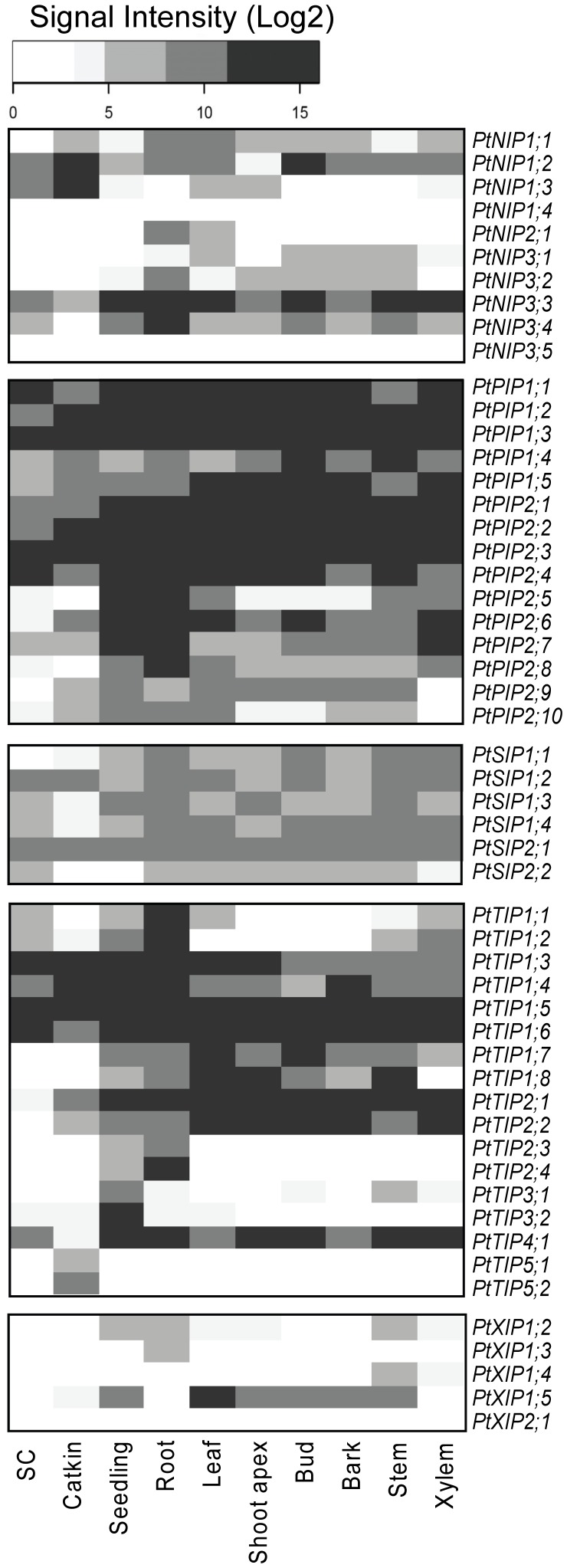
Expression profiles of AQP genes across tissues. Expression domains were computed from 110 “control” arrays (i.e. without “treatment” and “transgenic line” data). Arrays were normalised with GcRMA within each experiment. Each row of the heatmap corresponds to an AQP member. Color scale depicts maximal Log2 expression level. White represents below background level. Columns correspond to the eight sample types, namely SC for suspension cells (2 arrays: GSE16773, GSE17804), catkin (2 arrays: GSE13990), seedling (3 arrays: GSE13990), root (15 arrays: E-MEXP-1874, E-MEXP-2234, GSE13109, GSE13990, GSE16888, GSE16785, GSE17223, GSE17225, GSE19297), leaf (56 arrays: E-MEXP-1928, GSE9673, GSE13109, GSE13990, GSE14515, GSE14893, GSE15242, GSE16417, GSE16783, GSE16785, GSE17226, GSE17230, GSE21171, GSE24349, GSE27693, GSE16417), shoot apex (2 arrays: GSE16495, GSE21061), bud (14 arrays: GSE29335, GSE29336, GSE30320, GSE24349) bark (1 array: GSE29303), stem (4 arrays: GSE21480, GSE12152, GSE19467) and xylem (11 arrays: E-MEXP-2031, GSE13990, GSE16459, GSE20061, GSE27063, GSE3232). The number of arrays per tissue and the series accession numbers are given into brackets.

First insight of *AQP* functions in poplar was provided by an analysis of transcript accumulation in distinct tissues and organs sampled under control conditions (Figure 2). Organs/tissues were not equally represented in the data set ranging from one for bark to 56 for leaf samples. In some cases, a confounding effect cannot be excluded since sample types were collected on a unique *Populus* species. Biological inferences focused on positive signals, absence call reporting either absence of transcription or unsuitable experimental data.

Most *AQP* members were expressed in most vegetative tissues (Figure 2). Only *PtNIP1;4, PtNIP3;5*, and *PtXIP2;1* were called absent in all analysed tissues. *PIPs*, *SIPs* and *TIPs* exhibited higher expression levels than *XIPs* and *NIPs*. As can be expected, *AQP* expression pattern in suspension cells differed from multicellular tissues. The reproductive tissue showed the expression pattern the closest to that of suspension cells, both accumulating *PtNIP1;2* and *PtNIP1;3* transcripts but no *PtTIP1;7*/*PtTIP1;8* transcripts. *PtTIP5;1*/*PtTIP5;2* were preferentially expressed in mature catkins but not detected in floral bud nor in any vegetative organ. These results are in accordance with the predominant expressions of *AtTIP5;1, AtNIP4;1* and *AtNIP4;2* in Arabidopsis flowers and pollen [5], [41]. AtTIP5;1 has been suggested to be an urea transporter for pollen mitochondria and involved in nitrogen recycling in pollen tubes. Seedlings and roots were characterised by the broadest *AQP* expression patterns, with however some specific features. *PtTIP3* transcripts were strongly and preferentially accumulated in seedlings (Figure 2). In line, three out of the four *PtTIP3;1* EST were isolated from imbibed seeds and *TIP3* were reported as specific for maturating and dry seeds in several species [42]–[44]. In Arabidopsis, a high TIP3 protein abundance is maintained until completion of germination [45] and AtTIP3;1 and AtTIP3;2 are the only detectable TIPs in embryos during seed maturation and the early stages of seed germination [46]. Eight *AQPs* exhibited a root-preferred expression (Figure 2). Within three experiments (GSE17223/GSE17230, GSE13109 and GSE16783), transcript profiling was performed in both leaves and roots, enabling a straight comparison based on Log2ratio computation (Figure S2). Eleven *AQPs* were expressed at a higher level in roots than in leaves and only three *AQPs* exhibited a leaf-preferred expression. This analysis confirmed previously detected root-preferred expression of *PtNIP3;4, PtPIP2;8*, *PtTIP1;1/PtTIP1;2,* and *PtTIP2;3/PtTIP2;4*, and revealed new contrasts (*PtPIP2;2, PtPIP2;5, PtPIP2;7, PtTIP1;4* and *PtTIP4;1,* Figure S2). On the opposite, *PtPIP2;9, PtTIP1;8* and *PtXIP1;5* appeared consistently more expressed in leaves than in roots (Figure 2, Figure S2). The same expression patterns were reported for *PtPIP2;7, PtPIP2;8, PtTIP1;2, PtTIP1;8* and *PtTIP2;4*, based on *P. trichocarpa* samples analyzed on NimbleGen platform [12]. Similar expression patterns of *PtPIP2;8, PtPIP2;9* and *PtXIP1;5* were confirmed on *P. trichocarpa* using qPCR technology [47]. However, the cases of *PtPIP1;4/PtPIP1;`5, PtPIP2;3, PtTIP1;5/PtTIP1;6, PtTIP2;2* and *PtXIP1;2* highlighted that tissue-preferred expression may vary across genotypes (Figure S2).

Changes in plant *AQP* expression are known to occur during leaf development [8]. Although exhibiting growth-driven regulations, species and/or culture conditions affected the expression patterns of *PtNIP3;3/PtNIP3;4, PtTIP1;8* and *PtXIP1;5* (Figure S3). Meanwhile *PtPIP1;2*, *PtPIP2;6* and *PtTIP4;1* transcripts were accumulated in mature leaves while *PtPIP1;5, PtPIP2;2, PtPIP2;9, PtTIP1;4* and *PtTIP1;6* were preferentially-expressed in young leaves (Figure S3). Dealing with a woody species, the xylem transcriptome has been investigated. Except for the *PtXIP* subfamily, *AQP* expression patterns were relatively similar in xylem and aerial parts - leaf, shoot apex, bud, stem and bark (Figure 2). No *AQP* exhibited a xylem-preferred expression, consistently with the presence of this tissue in all organs (Figure 2). *PtPIP2;3/PtPIP2;4, PtTIP2;2* and *PtTIP4;1*, highly expressed in wood tissue, were more expressed in ray cambial cells as in fusiform cambial cells [48]. Interestingly PtPIP2;3, PtTIP2;2 and PtTIP4;1 proteins were detected in the plasma membrane of differentiating secondary vascular tissue [49].

### Comprehensive Analysis of Poplar *AQP* Expression Under Various Situations Reveals Heterogeneity in *AQP* Subfamilies Responsiveness and Co-regulations

Transcriptional regulations of plant *AQPs* are known to be isoform-specific [50]. Even within a subfamily, transcriptional responses clearly depend on the experimental procedures and vary across species as recently shown for *PIPs* in the case of drought stress [51]. In this context, a wide collection of experiments was analysed to highlight key features about poplar *AQP* responsiveness (Figure S4 to S8). To better address the questions of where and how consistent *AQP* expression was regulated, all transcriptional regulations - for each *AQP* gene under each of the 145 tested conditions- were compiled in Table 2. Given that some *AQPs* exhibited tissue- or organ-prefered expression (Figure 2), tissues and organs were considered separately. Based on a *post-hoc* grouping of common cues, 5 groups were delineated, namely abiotic stress, nutrional status, hormonal signalling, biotic interactions and temporal oscillation.

**Table 2:** Map of *AQP* responsiveness.

	Abiotic stress												Nutrition					Hormone				Biotic		Temporal oscillation				
	Water deficit			Osmoticum	Salt		R-hypoxia		L- wounding		Aluminium	Embolism, infiltration	Sarvation		Gln	Glc	Gln+Glc	CIM	SIM	MeJa	*GA*-modified	L-pathogen	Mycorrhiza	Seasonal			Diurnal	Diurnal
	L	X	R	R	X	R	R	L	L	R	R	X	L	B	B	B	B	St	Ca	SC	R	L	R	Lb	Fb	St		X
	40	1	6	1	2	3	3	1	3	1	3	2	6	3	2	2	2	2	2	1	5	4	2	3	4	1	18	4
*PtNIP1;1*	**x**	**C**	**2**	**C**	**1**	**x**	–	–	**2**	**C**	–	–	**2**	**1**	**1**	–	**C**	**1**	–	–	**1**	**2**	–	**1**	**3**	–	**x**	–
*PtNIP1;2*	**x**	–	**3**	**C**	–	–	**C**	–	**x**	–	–	–	**4**	–	–	–	–	–	–	–	**3**	**1**	–	**2**	–	–	**x**	–
*PtNIP1;3*	**1**	–	–	–	–	–	–	**C**	–	–	–	–	–	**1**	**C**	–	–	–	–	–	–	–	–	–	–	–	–	–
*PtNIP1;4*	–	–	–	–	–	–	–	–	–	–	–	–	–	–	–	–	–	–	–	–	–	–	–	–	–	–	–	–
*PtNIP2;1*	**x**	–	**4**	–	–	**2**	**x**	–	**2**	–	**1**	–	**x**	–	–	–	–	–	–	–	–	**1**	–	–	–	–	**x**	–
*PtNIP3;1*	**x**	–	–	–	–	**1**	–	–	**1**	–	–	–	–	**x**	**1**	–	**C**	**C**	–	–	–	–	–	–	–	–	–	–
*PtNIP3;2*	**x**	–	**1**	–	–	**1**	–	–	–	**C**	**1**	–	**x**	**2**	**C**	–	**C**	**C**	–	–	**1**	–	–	**C**	–	–	**1**	–
*PtNIP3;3*	**x**	**C**	**x**	**C**	**C**	**2**	**2**	–	**x**	–	–	**1**	**C**	**C**	–	–	–	**C**	–	**C**	**4**	**1**	**1**	**C**	–	–	**x**	**2**
*PtNIP3;4*	**x**	–	**x**	–	**1**	**C**	**2**	–	–	**C**	**1**	**1**	**2**	**C**	–	–	**1**	**1**	**C**	–	–	–	**1**	**C**	**3**	–	**7**	**2**
*PtNIP3;5*	–	–	–	–	–	–	–	–	–	–	–	–	–	–	–	–	–	–	–	–	–	–	–	–	–	–	–	–
*PtPIP1;1*	**x**	–	**3**	**C**	–	–	–	–	**x**	–	–	**1**	**2**	**1**	–	–	–	**x**	**1**	**C**	**1**	–	–	**C**	**3**	–	**7**	**1**
*PtPIP1;2*	**x**	**C**	–	**C**	**1**	**1**	**C**	–	**2**	–	**1**	**1**	**x**	**1**	–	–	–	**1**	**1**	**C**	**4**	**1**	**1**	**2**	**1**	–	**x**	**1**
*PtPIP1;3*	**x**	**C**	–	–	–	–	–	–	**x**	–	–	–	–	–	–	–	–	**C**	**1**	–	–	–	–	**1**	–	–	**5**	–
*PtPIP1;4*	**x**	**C**	**3**	**C**	**1**	**2**	–	–	–	**C**	**C**	**1**	**x**	**C**	**1**	–	**C**	**C**	**C**	–	–	–	**1**	**2**	**3**	–	**x**	–
*PtPIP1;5*	**x**	**C**	**4**	–	–	**2**	**2**	–	**1**	–	**1**	–	**x**	**C**	**C**	–	**C**	**x**	–	–	–	**1**	–	–	**2**	–	**x**	**1**
*PtPIP2;1*	**x**	–	**1**	**C**	**C**	–	**2**	–	**1**	–	**1**	**1**	–	**2**	–	–	**1**	**C**	–	–	–	–	–	**x**	**3**	–	**x**	–
*PtPIP2;2*	**x**	–	**3**	**C**	**C**	**x**	**1**	–	**1**	–	**1**	–	**3**	–	–	**1**	**1**	**C**	**1**	–	–	**3**	**1**	**C**	**2**	**C**	**x**	–
*PtPIP2;3*	**x**	**C**	–	–	**1**	–	**C**	–	**1**	–	**1**	–	**3**	**2**	**1**	–	**1**	**C**	–	**C**	**1**	**1**	–	**C**	–	–	**x**	–
*PtPIP2;4*	**x**	–	**2**	**C**	**1**	–	–	–	**x**	–	**2**	**C**	**x**	**2**	–	–	**1**	–	–	–	**4**	**1**	–	**2**	**x**	**C**	**x**	–
*PtPIP2;5*	**x**	**C**	**4**	–	**1**	**2**	–	–	**2**	–	**1**	**1**	**2**	**2**	**1**	–	**C**	**x**	**1**	–	**C**	–	**1**	–	–	**C**	**x**	**1**
*PtPIP2;6*	**x**	**C**	–	**C**	**1**	**1**	–	–	**2**	–	**1**	**1**	**4**	**1**	–	–	**1**	**x**	**C**	**C**	**3**	**2**	–	**x**	**2**	–	**x**	**1**
*PtPIP2;7*	**x**	**C**	**C**	**C**	**1**	**2**	**C**	–	**1**	–	**x**	**C**	**3**	**x**	**1**	–	–	**1**	**C**	–	**C**	**1**	**1**	**x**	**1**	–	**x**	**1**
*PtPIP2;8*	**x**	–	**5**	**C**	**1**	–	**C**	–	**x**	–	–	–	**2**	**2**	**C**	–	**C**	**1**	**C**	–	**4**	–	–	**C**	**1**	–	**x**	**1**
*PtPIP2;9*	**x**	–	–	–	–	–	–	–	**1**	–	–	–	**3**	**1**	–	–	**1**	**C**	**C**	–	–	**2**	–	**C**	**3**	–	**x**	–
*PtPIP2;10*	**x**	–	**5**	**C**	–	**x**	**C**	–	**x**	**C**	**1**	–	**2**	**2**	–	**x**	**1**	**1**	**1**	–	**2**	**1**	**1**	–	**1**	**C**	**x**	–
*PtSIP1;1*	–	–	–	**C**	–	–	–	–	–	–	–	–	–	**C**	–	–	–	–	–	–	–	–	–	**C**	**3**	–	–	–
*PtSIP1;2*	**x**	**C**	**3**	–	**C**	–	**2**	–	**2**	–	**1**	**1**	**1**	**C**	–	–	–	–	–	–	**2**	**1**	–	**C**	**1**	–	**x**	–
*PtSIP1;3*	**x**	**C**	**2**	–	**1**	**1**	**2**	–	**1**	–	–	–	–	**C**	**1**	–	**1**	**1**	–	–	–	–	**1**	**C**	**1**	**C**	**x**	–
*PtSIP1;4*	**x**	–	–	**C**	–	**1**	**1**	–	–	–	–	–	**x**	–	**C**	–	**C**	**1**	**C**	–	–	–	**1**	**1**	**1**	–	**3**	–
*PtSIP2;1*	**x**	**C**	–	**C**	–	–	**1**	**C**	**2**	**C**	–	–	–	**1**	–	–	**C**	**x**	**C**	–	–	**2**	–	**C**	**1**	–	**x**	–
*PtSIP2;2*	–	–	**2**	–	–	–	**x**	–	–	–	–	–	**1**	–	–	–	–	**x**	**C**	**C**	**2**	**1**	–	–	–	–	–	–
*PtTIP1;1*	**x**	**C**	**4**	**C**	**1**	**C**	**C**	–	**2**	**C**	–	**1**	**3**	–	**1**	**1**	**1**	**1**	**C**	–	**C**	–	**x**	–	–	**C**	**x**	**2**
*PtTIP1;2*	–	**C**	**5**	**C**	**1**	**C**	**C**	–	–	–	**1**	**C**	–	–	–	–	–	–	–	–	**C**	–	**x**	–	–	**C**	–	**2**
*PtTIP1;3*	**x**	–	**C**	–	–	**C**	**2**	–	**x**	–	–	**1**	**4**	**x**	–	–	**C**	**C**	**C**	**C**	**C**	**1**	**1**	**C**	**2**	–	**14**	**2**
*PtTIP1;4*	**x**	**C**	**C**	**C**	**1**	**C**	–	–	**x**	–	–	**C**	**5**	**C**	**1**	–	**1**	**C**	**C**	–	**C**	**1**	**x**	–	**3**	–	**x**	**2**
*PtTIP1;5*	**x**	**C**	**1**	**C**	**1**	**2**	**C**	–	**x**	–	–	–	**3**	**C**	**C**	**1**	**C**	**C**	**1**	–	**1**	**2**	**1**	**2**	**1**	–	**x**	**1**
*PtTIP1;6*	**x**	**C**	–	–	**1**	**1**	**C**	–	**2**	–	–	–	**4**	**C**	**1**	–	–	**C**	–	**C**	**2**	**3**	**1**	**1**	**1**	–	**x**	–
*PtTIP1;7*	**x**	–	**1**	**C**	–	–	**2**	–	**2**	–	–	**1**	**3**	**1**	–	–	–	**C**	**1**	–	**C**	**1**	–	**C**	**1**	**C**	**x**	–
*PtTIP1;8*	**x**	–	–	–	–	**2**	**C**	–	**C**	–	**2**	–	**5**	**C**	**C**	**1**	**C**	**C**	**x**	–	**C**	**1**	**1**	**C**	**1**	–	**x**	–
*PtTIP2;1*	**x**	–	**C**	**C**	–	**C**	**1**	–	**1**	–	–	**1**	**x**	**2**	**C**	–	–	**C**	**1**	–	**1**	**3**	**1**	**C**	–	–	**x**	–
*PtTIP2;2*	**x**	**C**	**C**	**C**	**1**	–	–	–	**x**	**C**	**1**	–	**1**	**C**	**C**	–	**1**	**C**	**1**	–	**1**	**1**	–	**x**	–	–	**x**	**2**
*PtTIP2;3*	–	–	**4**	**C**	–	**1**	**1**	–	–	–	–	–	–	–	–	–	–	–	–	–	**C**	–	–	–	–	–	–	–
*PtTIP2;4*	–	–	**4**	**C**	–	**C**	**1**	–	–	–	**1**	–	–	–	–	–	–	–	–	–	**C**	–	**1**	–	–	–	–	–
*PtTIP3;1*	–	–	–	–	–	–	–	–	–	–	–	–	**1**	–	–	–	–	–	–	**C**	–	–	–	–	–	–	–	–
*PtTIP3;2*	**x**	–	–	–	–	–	–	–	–	–	–	–	–	–	–	–	–	–	–	–	–	–	–	–	–	–	**1**	–
*PtTIP4;1*	**x**	**C**	**1**	–	**1**	**C**	**C**	–	**x**	–	–	**1**	**3**	**C**	–	–	–	**C**	**x**	–	–	**1**	–	**C**	**1**	–	**x**	–
*PtTIP5;1*	–	–	–	–	–	–	–	–	–	–	–	–	–	–	–	–	–	–	–	–	–	–	–	–	–	–	–	–
*PtTIP5;2*	–	–	–	–	–	–	–	–	–	–	–	–	–	–	–	–	–	–	–	–	–	–	–	–	–	–	–	–
*PtXIP1;2*	–	**C**	**C**	**C**	–	**2**	–	–	**1**	–	**1**	–	–	–	–	–	–	–	–	–	–	–	**C**	–	–	–	–	–
*PtXIP1;3*	–	–	**4**	–	–	–	–	–	–	–	–	–	–	–	–	–	–	–	–	–	–	–	–	–	–	–	–	–
*PtXIP1;4*	–	–	–	–	–	–	–	–	–	–	–	–	–	–	–	–	–	–	–	–	–	–	–	–	–	**C**	–	–
*PtXIP1;5*	**x**	–	–	–	–	–	–	–	**2**	–	–	–	**x**	–	–	–	**C**	**C**	**C**	–	–	**2**	–	**C**	**1**	**C**	**x**	–
*PtXIP2;1*	–	–	–	–	–	–	–	–	–	–	–	–	–	–	–	–	–	–	–	–	–	–	–	–	–	–	–	–

Regulations of *AQP* expression in response to distinct cues are depicted in distincft organs and tissues: leaf (L), root (R), xylem (X), bark (B), stem (St), floral bud (Fb), leaf bud (Lb) and suspension cell (SC). The number of experiments analysed is given in the heading. Responsiveness is described as follow: “−” denotes absence of regulation (FC<1.5), “C” denotes consistent regulations (FC≥1.5, 100% cases), a number denotes intermediary cases (FC≥1.5, number of cases). A cross indicates interaction: both down- and up-regulations are observed within a category (FC≥1.5).

### Transcriptional Regulation of *AQP* Expression Accompanying Abiotic Challenges

As expected for a model plant of agronomic interest, water deficit was the most studied abiotic stress (Table 2). While about one third of *AQPs* were not responsive to water deficit, *PtPIP1;2* and *PtPIP2;7* expressions were consistently up-regulated in all organs (Table 2, Figure S4A). Identified as preferentially expressed in roots under control conditions (Figure 2), *PtPIP2;8*, *PtTIP1;2* and *PtTIP2;3/PtTIP2;4* were even more expressed in roots under water deficit. In addition, *PtTIP2;2* expression was one of the strongest water deficit-induced up-regulations in roots while expressions of *PtNIP2;1*, *PtPIP2;2* and *PtPIP2;10* were down-regulated. Osmotic stress and soil water deficit induced similar patterns of *AQP* regulation in Soligo root apices except for *PtNIP1;1* and *PtXIP1;2*. Drought-driven regulations occurring in leaves were found to be mostly inconsistent across the 40 comparisons, reflecting either wide genotype diversity or large number of experiments (Table 2, Figure S4A). Accordingly, drought-driven transcriptome response in leaf has been shown to be shaped by time of day, to be dependent on genotype x treatment interaction and on clone history [27], [29], [52]. However the strongest drought responses were down-regulations of *PtPIP1;5, PtPIP2;9*, *PtTIP1;6*, *PtTIP1;8* and *PtXIP1;5* expressions (Figure S4A). The highest up-regulations of expression were found for *PtTIP1;1* and *PtTIP1;4* in xylem and for early response in leaf of a drought-tolerant genotype (Figure S4A). Using qPCR approaches, similar drought responses have been reported in poplar leaves for *PtPIP1;2, PtPIP2;7* and *PtXIP1;5* [23], [24]. While some AQPs have been suggested as playing a role in regulation of leaf hydraulics with a possible link to stomatal conductance and drought tolerance, such as PIP2;5 orthologs [24], the above-cited *AQPs* could be considered as drought markers.

Salt and hypoxia-driven responses were similar in roots, both stresses repressing several *AQP* expressions (Figure S4B). However the accumulation of *PtPIP2;10* transcripts seemed to be a key *AQP* signature of hypoxia in roots, but this result has to be confirmed in other *Populus* species. The impact of wounding on *AQP* patterns was strong but clearly dependent on both leaf plastochron index and time after treatment, thus precluding general conclusion (Figure S4C). In leaves collected on tree submitted to nitrogen limitation, expression of several *AQPs* (*PtNIP3;3/PtNIP3;4, PtPIP2;3, PtPIP2;5/PtPIP2;6, PtPIP2;7, PtTIP1;1, PtTIP1;3/PtTIP1;4* and *PtTIP4,1*) tended to be up-regulated while the expression of some others (*PtNIP2;1, PtPIP2;2, PtPIP2;9/PtPIP2;10, PtTIP1;5/PtTIP1;6, PtTIP1;7/PtTIP1;8* and *PtXIP1;5*) was down-regulated under prolonged starvation (Figure S4D). In lines, incubation in water of partially defoliated stem led to the accumulation of *PtNIP3;3/PtNIP3;4, PtPIP2;5/PtPIP2;6* and *PtTIP4;1* transcripts and to reduced expression of *PtTIP1;5/PtTIP1;6* and *PtTIP1;8* in bark. In addition, expressions of *PtSIP1;1/PtSIP1;2* and *PtSIP1;3* were up-regulated in bark of starved stem only (Table 2). In response to starvation, *PtTIP1;4* showed a contrasting response in bark and leaf tissues (Figure S4D). Given that these transcriptional regulations were mostly reversed – or alleviated - when incubation media included glutamine (in combination or not with glucose) and that glucose feeding did not modify *AQP* expression (Table 2), these *AQPs* appeared to be responsive to nitrogen status.

### Transcriptional Regulation of *AQP* Expression in Response to Other Cues

As previously observed in other species [53], poplar *AQPs* were also diversely responsive to modification of hormonal status (Table 2). Distinct phases of poplar micro-propagation induced large modifications of *AQP* expression (Figure S5A). Biotic interactions were also accompanied with transcriptional regulation of *AQP* expression (Table 2). In line with its root-preferred expression under control conditions (Figure 2), *PtXIP1;2* expression was strongly induced by mycorrhization (Figure S6A). PtXIP1;2 being apparently devoid of water transport in *Xenopus leavis* oocyte [23], it suggests another role during mycorrhizal interactions, e.g urea or ammonium transport [3]. Meanwhile enhanced expression of root *AQPs* and increased root hydraulic conductivity was shown in ectomycorrhizal seedlings of *P. tremula* × *P. tremuloides* [18] and root hydraulic conductivity of balsam poplar (*P. balsamifera*) was differentially enhanced regarding mycorrhizal fungal species [54]. All in all, mycorhizal fungi appear to interfere with the aquaporin-mediated transport, whatever the transported substrate, as suggested for *P. angustifolia* [55]. As previously reported during infection by *Melampsora larici-populina* incompatible strain (PICME technology, [56]), foliar infection by another biotrophic fungi also repressed the expression of *PtTIP1;5/PtTIP1;6,* and *PtPIP2.2* (Figure S6B). In soybean, 24 of 32 *AQP* genes were down-regulated in the hours following infection by avirulent *Pseudomonas syringae* [57]. These crosstalks suggest that controlling water movement could be a mechanism of pathogen inhibition during plant defense.

Seasonal variations of *AQP* expression were recorded in different organs of this perennial species (Table 2, Figure S7). The only regulation of the stem-preferred *PtXIP1;4* was detected during winter hardening, opening new hypotheses. As in other species [58], [59], poplar *AQP* expression generally varied around the day (Figure S7B). Evidence of diurnal regulation was found, even if *AQP* expressions were often inconsistent across genotypes (Table 2). Nevertheless, *PtPIP1;1, PtPIP2;3* and *PtPIP2;7* were more expressed during dark period than during light period in leaf and xylem while *PtPIP2;9* and *PtXIP1;5* were clearly light induced in leaf (Figure S7B), indicating different physiological functions for these AQP members. Transcriptional regulations of *AQPs* co-occurring with the over-expression or silencing of several genes in distinct transgenic lines are not detailed here (see Figure S8). Result show that transgenic lines constitute a source of diversity, disturbing *AQP* expression.

### Member-specific Expression Profiles

**Figure 3:**
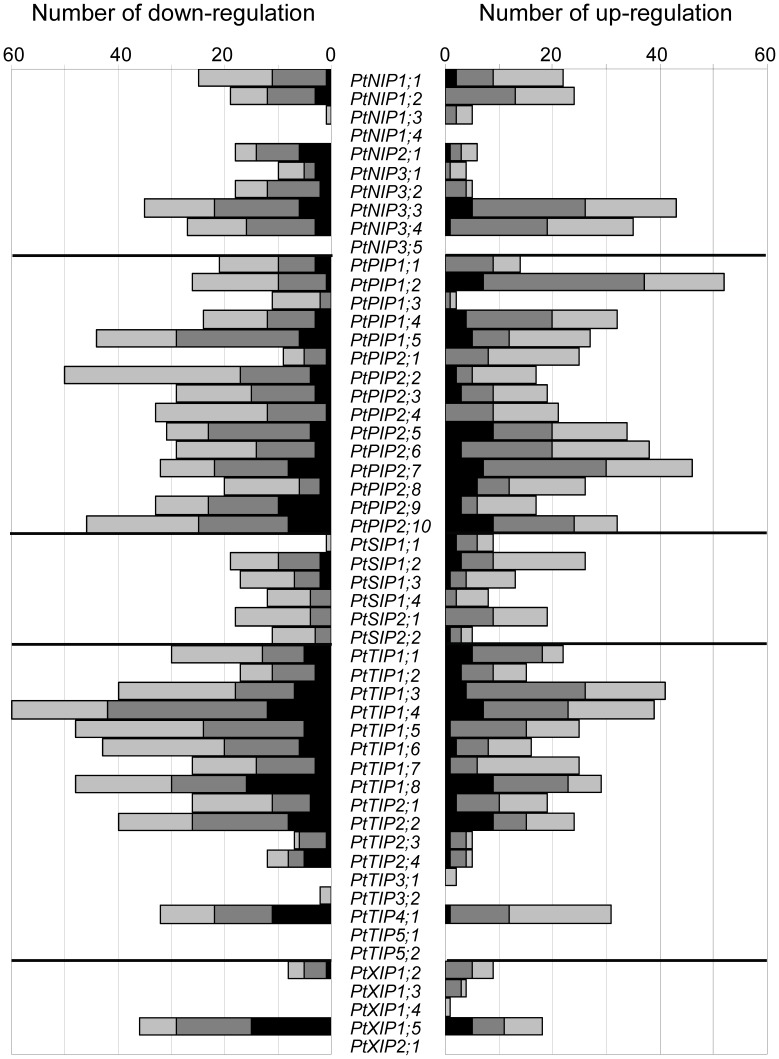
Distribution of regulations by class of fold-change. For each AQP, up- and down-regulations were counted across 145 comparisons and classified according to the fold change (FC) level: weak regulation 1.5≤FC<2 (light grey); 2≤FC<4 moderate regulation (grey) and FC≥4 strong regulation (dark grey).

The expression of five *AQPs* was never regulated (Figure 3). Among those, our meta-analysis show no evidence of expression for *PtNIP1;4, PtNIP3;5* and *PtXIP2;1*, suggesting very narrow expression patterns (Figures 2 and 3). The functionality of these genes may also be questioned since no corresponding EST has been reported to date in GenBank, and no clear evidence of their transcription was found in another genome-wide transcript profiling based on an independent platform [12]. While never regulated, *PtTIP5;1*/*PtTIP5;2* expressions were restricted to mature catkins under control conditions (Figure 2, Figure S3) and their responsiveness within catkins could be suspected but not tested.

While expressed under control conditions, six *AQPs* were found to be hardly responsive to tested cues (Figure 3). *PtNIP1;3* was mostly expressed in catkins and was only punctually regulated. *PtXIP1;3* was found exclusively expressed in roots but could be expressed in other organs [12]. *PtXIP1;4* was expressed in stem and to a lesser extent in xylem in accordance with literature [12], [15]. *PtTIP3;1/PtTIP3;2* exhibited a seedling-preferred expression, but their transcripts have been previously detected in other organs [12]. Given that *PtPIP1;3* was constitutively expressed at high level in all organs under control conditions but underwent only few and weak down-regulations, the absence of responsiveness was not linked to the wideness nor the intensity of expression under control conditions.

Concerning *AQPs* exhibiting numerous transcriptional regulations, *PIP* and *TIP* members were more frequently regulated than those of other subfamilies but regulations of expression were mainly of moderate intensity (fold-change ≤4, Figure 3). *PtTIP1;4* was found to be the most responsive gene, regulated in almost 100 over 145 comparisons (Figure 3, Table 2). Expressions of *PtTIP1;8* and *PtXIP1;5* occurred preferentially in leaves, were strongly regulated (fold-change ≥4) and were responsive to many cues. *XIP1;5* was recently found ubiquitously expressed [23], disagreeing with our results, *i.e.* absence of expression in roots as well as absence of root EST. More interestingly PtXIP1;5 was shown to function as water transporter in *Xenopus leavis* oocyte [23]. Beside, the strong regulation of *PtXIP1;5* expression in the ProHSP:*FT* lines was not found under constitutive over-expression of *FT1* and *FT2* (Figure S8C), suggesting a potential response to heat induction, which is consistent with its demonstrated drought sensitivity [23].

### Divergence and Redundancy of *AQP* Duplicates

**Figure 4:**
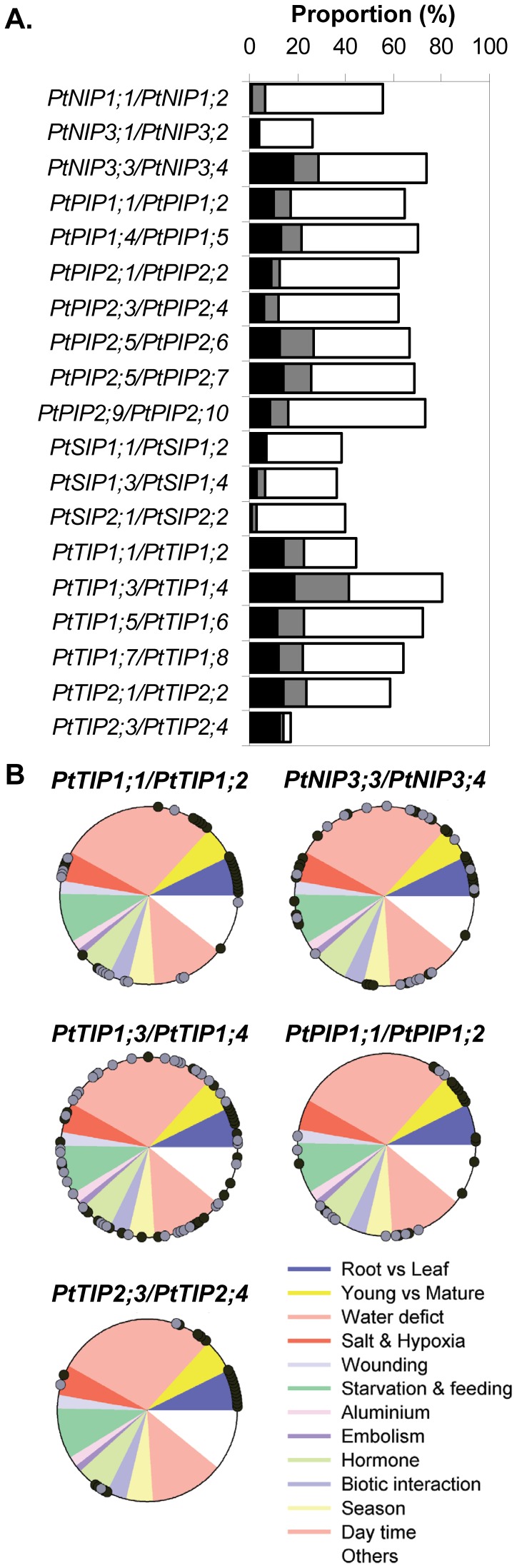
Occurrence of convergent regulation within gene pairs. **A. Proportion of convergent versus divergent regulations.** Percent of comparison in which duplicates underwent convergent regulations is shown in grey (1.5≤FC<2) or in black (FC≥2). White bar indicates the proportion of comparison inducing divergent regulation of expression. **B. Repartition of convergent regulation over experimental categories within five gene pairs**. Each dot indicates that gene duplicates underwent convergent regulations under one comparison. Experimental categories are depicted by colour sectors. Dot colour denotes fold change level, black: FC≥2 and grey: 1.5≤FC<2.

Our meta-analysis gave access to co-expression patterns of *AQP* members. To test whether duplicates were functionally redundant, their regulation patterns were pair-wise compared (Figure 4). For each *AQP* pair, we determined the percentage of comparisons for which none of the duplicates underwent transcriptional regulation. Varying from 20% (*PtTIP1;3/PtTIP1;4*) to 80% (*PtTIP2;3/PtTIP2;4*), this proportion reflects the wideness of gene pairs expression and the over-representation of studies carried on leaf (Figure 4A). Then, we determined the percentage of comparisons for which a divergence in response was observed, i.e. either only one member of the pair being regulated or both oppositely regulated. In *Arabidopsis*, AtPIP2;2/AtPIP2;3 shared a high structural similarity and were found to be functionally divergent on the basis of distinct expression properties [60]. Such divergent responses concerned more than 50% of regulation events for all gene pairs, except for *PtTIP2;3/PtTIP2;4* (Figure 4A). The latter exhibited similar expression patterns under control conditions (being preferentially expressed in root and seedling, Figure 2), were responsive to few cues and shared convergent responses to modification of gibberellin status and to water deficit (Table 2, Figure 4). These results suggest functional redundancy of these paralogs. While exhibiting balanced proportion of convergent and divergent regulations, convergent regulations of *PtTIP1;1/PtTIP1;2* were mainly observed in comparisons carried on root and xylem, suggesting a putative functional redundancy in these organs (Figure 4B). In contrast, convergent regulations of *PtTIP1;3/PtTIP1;4* expression were observed in 51% cases but occurred over a large panel of cues, organs and species. This random distribution precluded concluding about functional redundancy but indicated that this gene pair encodes generic *AQPs*. For some gene pairs, convergent regulations were observed in response to specific cues. For instance, most convergent regulations of *PtPIP1;1/PtPIP1;2* expression were observed in response to leaf maturity, hormonal treatment and day time (Figure 4B). Both genes were strongly expressed in leaves and their expressions were commonly enhanced during leaf aging. Concerted regulations of *PtNIP3;3/PtNIP3;4* expression mainly occurred in response to abiotic stresses and day time. Co-regulation within five gene pairs was very scarce (less than 20% of regulation events) whatever the responsiveness of the pairs, indicating clear functional divergence between duplicates. In bream and salmon respectively, two and three functional *AQP* paralogs were differentially distributed and regulated in the intestinal epithelium [61], [62]. These results suggested a fine regulation of transcellular transport in regards to regulation of *AQP* paralogs. The divergence between duplicates could be partly due to the larger responsiveness of one duplicate as compared to its counterpart (for instance, see *PtSIP1;1/PtSIP1;2,* Figure 3). Globally, the convergence level was slightly higher for the *PtTIP* pairs than for the *PtPIP* pairs, especially if absence of regulation of the two pair members is considered as convergence too (Figure 4A). Then residual functional redundancy may have been conserved at a higher level in the *PtTIP* subfamilly than in the *PtPIP* subfamilly. In addition, most *PtTIP* pairs expression patterns showed higher tissue-specificity than those of *PtPIPs* (Figure 2), suggesting that TIPs could more contribute to cell identity than PIPs.

### Conclusions

While considered as molecular entry into plant water relations, diversity of AQP functions in plants together with family amplification make their characterisation challenging. As a step towards a better understanding of transcriptional regulation, this meta-analysis of all Affymetrix data publicly available has provided a comprehensive picture of poplar *AQP* expression and regulation at the whole family scale. Through a detailed confrontation with literature, our results were globally validated by previously published information on *AQP* expression and regulation. In the meantime, gathering usually un-compared cues (for instance biotic *vs* abiotic) provided novel information. The responsiveness of all genes to a given cue as well as the impact of many cues on the expression of each member were provided without *a priori*, revealing key features but also highlighting the strong functional divergence within the *AQP* family.

## Supporting Information

Figure S1 Analysis of “sibling” probe sets reveals the sensitivity of median-based procedure for the automatic extraction of gene-centred information in the case of multi-experiment comparison.
(PDF)

Figure S2 Root- and leaf-preferred expression of *AQPs.*
(PDF)

Figure S3 Young leaf- and mature leaf-preferred expression of *AQPs.*
(PDF)

Figure S4 Transcriptional regulation of *AQP* expression under abiotic challenges.
(PDF)

Figure S5 Transcriptional regulation of *AQP* expression in response to hormonal signalling.
(PDF)

Figure S6 Transcriptional regulation of *AQP* expression under biotic interactions.
(PDF)

Figure S7 Seasonal or diurnal regulation of *AQP* expression.
(PDF)

Figure S8 Differential *AQP* expression in response to other cues.
(PDF)

Table S1 Listing of *Aquaporin*-targeting probe sets. The table details for each Affymetrix probe set, its ID, the tools enabling its detection (see “materials and methods” and Figure 1), its target gene ID and its annotation.
(XLS)

Table S2 Number of probe sets per *AQP* member.
(XLS)
