# Supplementary material for: Developmental and Environmental Regulation of Aquaporin Gene Expression across Populus Species: Divergence or Redundancy?
Source: PLoS One. 2013 Feb 5;8(2):e55506. doi: 10.1371/journal.pone.0055506 (PMC3564762; doi:10.1371/journal.pone.0055506)
Supplement: Figure S1 — Analysis of “sibling” probe sets reveals the sensitivity of median-based procedure for the automatic extraction of gene-centred information in the case of multi-experiment comparison. (PDF) [file pone.0055506.s001.pdf]

**Figure S1. Analysis of "sibling" probe sets reveals the sensitivity of median-based procedure for the automatic extraction of gene-centred information in the case of multi-experiment comparison**. Twenty-two *AQP* genes are represented by several probe sets on the Poplar Affymetrix Gene chip. (**A**) Correlations between median-based and maximum-based evaluation of gene regulation (Log2 ratio). Median values were computed without preliminary filtering (i.e. by considering all, even null, Log2 ratios). For genes targeted by two probe sets, both procedures (median or maximum) gave similar results as shown by the high regression coefficient. However, the divergence from 1:1 relationship which is observed in most but not all cases questioned the quality of both procedures for the assessment of gene regulation. As expected, median provided lower values than maximum-based procedure. For genes targeted by three or more probe sets, the discrepancy between the two estimates was strongly exacerbated in most cases. (**B**) We hypothesised that the discrepancy between estimates could reflect differential probe set suitability for expression profiling across a wide range of experiments (including several *Populus* species). For a given *AQP* gene, probe sets were screened on the basis of their signal values across all arrays. Median was recalculated by taking into account only P-called probe sets (P/A calls based on background set at 3.2, see Cohen *et al*., 2010, for details). This filtering enhanced correlation between estimates, thus highlighting that median-based procedure required to be coupled to P/A calls filtering to be a suitable estimator of gene regulation. Meanwhile, P/A call procedure can hardly be automatically run in the case of complex matrices of experiments. This point is clearly illustrated in (**C**). Out of the six probe sets targeting *PtPIP2;4*, Ptp.436.1.S1_at was automatically filtered out. This probe set, which was detected using all *in silico* tools (Fig. 1), was designed in UTR region but had no perfect match with reference sequences (Table S1). The five remaining probe sets exhibited strongly contrasted profiles across experiments in a species-specific manner. In such case, P/A call cannot be used as filter as probe sets suitability varies along the whole matrix.

The use of median as an estimator of gene expression/regulation across multiple experiments clearly induced a bias. Median procedure requires the suitability of all "sibling" probe sets for any genotype/species, in order not to dilute signal intensity with null or weak signal. Maximum-based method appears more appropriate than median in the context of meta-analyses using a multispecies-designed array. Using maximum estimator does not depend on the number of probe set, nor require manual curation in complex matrices and nor dilute signal intensity (which depends on both the number of sibling probe set per gene and on the number of suitable probe set per gene per genotype).

| | (A) Correlation parameter between median and maximal estimators (without filtering) | | (B) Correlation parameter between median and maximal estimators (after only one probe set filtered out ) | |
|---|---|---|---|---|
| **Two probe sets** | **$R^2$** | **a** | **$R^2$** | **a** |
| *PtTIP3;1* | 1 | 0.50 | | |
| *PtTIP1;2* | 1 | 0.96 | | |
| *PtTIP1;4* | 0.99 | 0.88 | | |
| *PtPIP2;7* | 0.99 | 0.51 | | |
| *PtTIP2;2* | 0.99 | 0.53 | | |
| *PtTIP1;3* | 0.97 | 0.55 | | |
| *PtPIP1;4* | 0.98 | 0.51 | | |
| *PtXIP1;2* | 0.96 | 1.0 | | |
| *PtNIP1;2* | 0.96 | 0.53 | | |
| *PtTIP1;7* | 0.93 | 0.73 | | |
| **Three probe sets** | | | | |
| *PtPIP2;2* | 0.98 | 0.85 | | |
| *PtPIP2;9* | 0.91 | 0.69 | | |
| *PtTIP1;5* | 0.91 | 0.76 | | |
| *PtTIP1;8* | 0.89 | 0.70 | 0.98 | 0.85 |
| *PtPIP1;2* | 0.84 | 0.63 | 0.89 | 0.78 |
| *PtPIP2;10* | 0.84 | 0.71 | 0.94 | 0.80 |
| *PtNIP3;4* | 0.82 | 0.59 | 0.96 | 0.78 |
| *PtPIP1;3* | 0.24 ns | - | 0.93 | 0.52 |
| *PtSIP1;2* | 0.52 ns | - | 0.79 | 0.49 |
| **Four probe sets** | | | | |
| *PtNIP3;3* | 0.93 | 0.78 | | |
| **Six probe sets** | | | | |
| *PtPIP2;1* | 0.81 | 0.32 | 0.89* | 0.67 |
| *PtPIP2;4* | 0.34 ns | - | 0.67* | 0.31 |
| | | | | |
| (ns): not significant *P*-value >0.05 - (*) two probe sets filtered out | | | | |

**(C)**

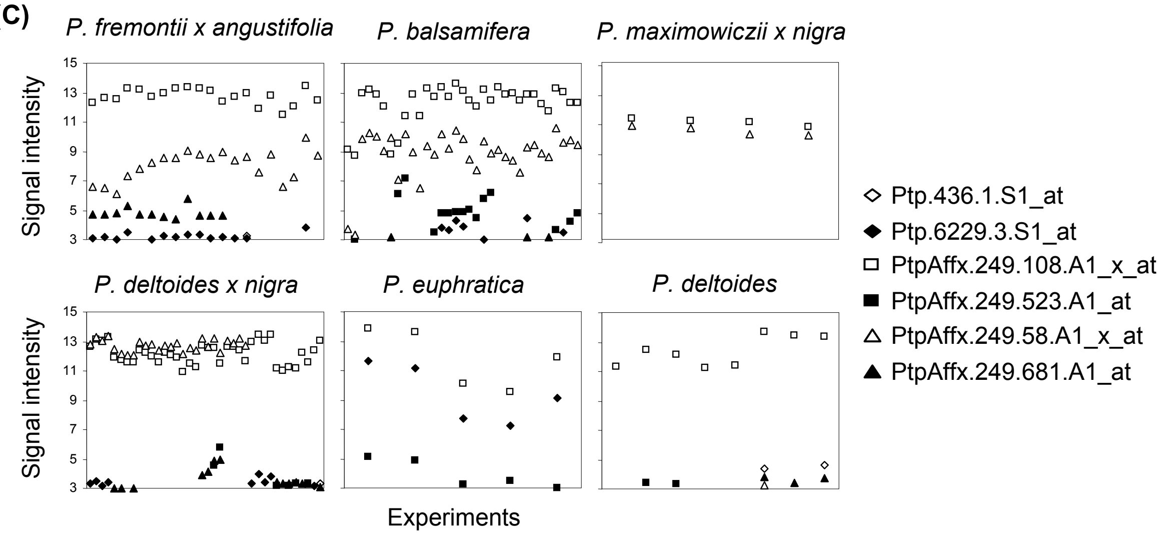


**Figure S1**
