# Supplementary material for: Developmental and Environmental Regulation of Aquaporin Gene Expression across Populus Species: Divergence or Redundancy?
Source: PLoS One. 2013 Feb 5;8(2):e55506. doi: 10.1371/journal.pone.0055506 (PMC3564762; doi:10.1371/journal.pone.0055506)
Supplement: Figure S2 — Root- and leaf-preferred expression of AQPs. (PDF) [file pone.0055506.s002.pdf]

Figure S2. Root- and leaf-preferred expression of *AQP*. Difference of expression level is indicated as Log2 ratio of root relative to leaf samples. Differential *AQP* transcript accumulations between organs were hierarchically clustered using Euclidean distance. Each row of the heatmap corresponds to an *AQP* gene. Color scale depicts Log2ratio value: Green represents leaf-preferred expression and red represents root-preferred expression. Columns correspond to 12 comparisons between root and leaf samples of distinct genotypes (*Populus canescens, P. fremontii x P. angustifolia* RM5*, P. deltoides x P. nigra* Carpaccio and Soligo as indicated). Samples were collected from control trees grown in hydroponics (1, 2 and 4), in soil without water limitation (5 and 11), 90h after leaf mechanical wounding (3), 36h after irrigation stop (short-turn drought, 6 and 12), after 10-day mild drought (soil relative extractable water at 20 – 35%: 7 and 9) or after 10-day moderate drought (soil relative extractable water at 10 – 20%: 8 and 10). Microarray data were found under the series accession numbers: GSE13109 (1 and 2), GSE16786 (3 and 4), GSE17223 and GSE17230 (5 to 12).

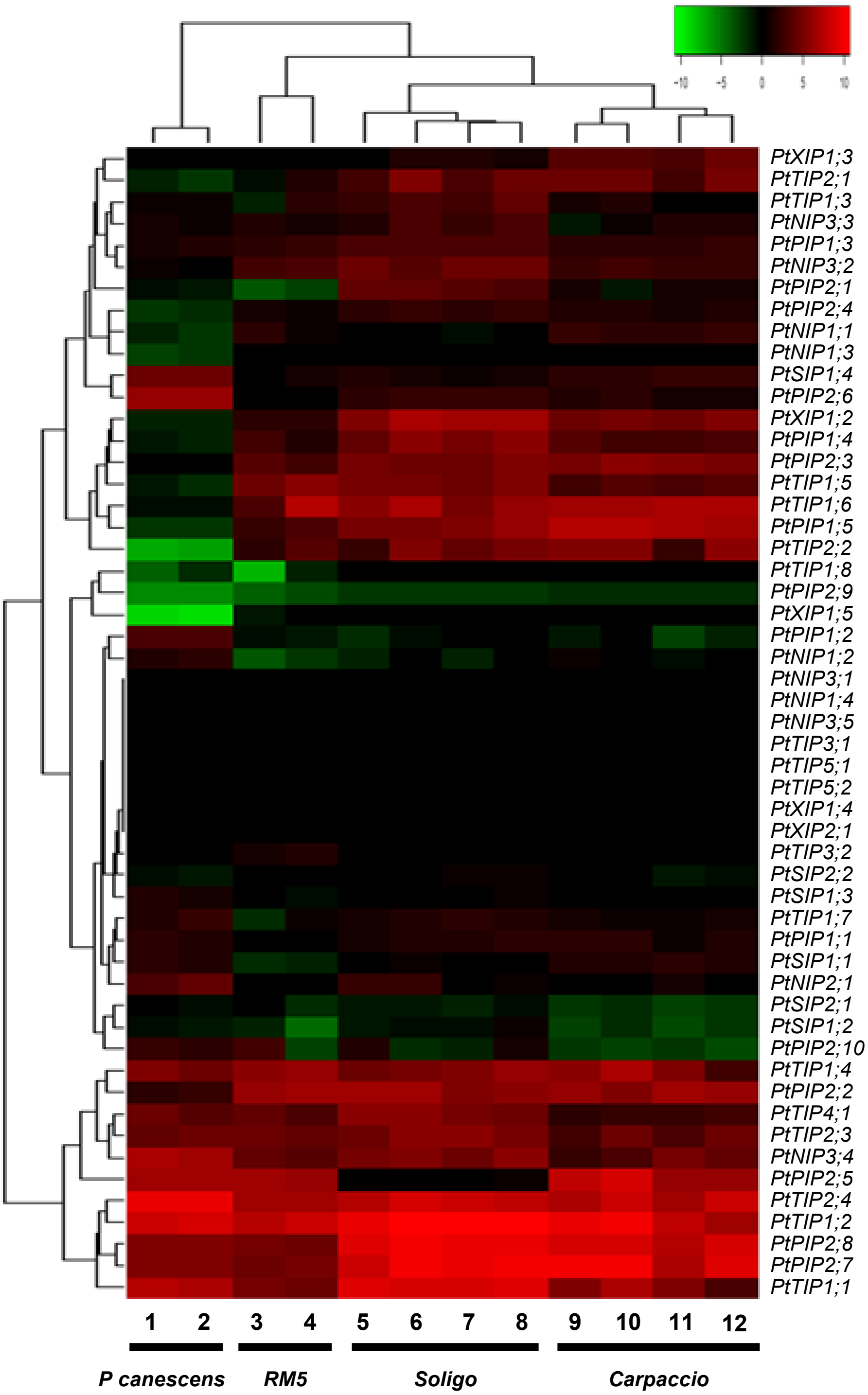

-10
-5
0
5
10
PtXIP1;3
PtTIP2;1
PtTIP1;3
PtNIP3;3
PtPIP1;3
PtNIP3;2
PtPIP2;1
PtPIP2;4
PtNIP1;1
PtNIP1;3
PtSIP1;4
PtPIP2;6
PtXIP1;2
PtPIP1;4
PtPIP2;3
PtTIP1;5
PtTIP1;6
PtPIP1;5
PtTIP2;2
PtTIP1;8
PtPIP2;9
PtXIP1;5
PtPIP1;2
PtNIP1;2
PtNIP3;1
PtNIP1;4
PtNIP3;5
PtTIP3;1
PtTIP5;1
PtTIP5;2
PtXIP1;4
PtXIP2;1
PtTIP3;2
PtSIP2;2
PtSIP1;3
PtTIP1;7
PtPIP1;1
PtSIP1;1
PtNIP2;1
PtSIP2;1
PtSIP1;2
PtPIP2;10
PtTIP1;4
PtPIP2;2
PtTIP4;1
PtTIP2;3
PtNIP3;4
PtPIP2;5
PtTIP2;4
PtTIP1;2
PtPIP2;8
PtPIP2;7
PtTIP1;1
1 2 3 4 5 6 7 8 9 10 11 12
P canescens
RM5
Soligo
Carpaccio

Figure S2
