# Supplementary material for: Developmental and Environmental Regulation of Aquaporin Gene Expression across Populus Species: Divergence or Redundancy?
Source: PLoS One. 2013 Feb 5;8(2):e55506. doi: 10.1371/journal.pone.0055506 (PMC3564762; doi:10.1371/journal.pone.0055506)
Supplement: Figure S3 — Young leaf- and mature leaf-preferred expression of AQPs. (PDF) [file pone.0055506.s003.pdf]

Figure S3. Young leaf- and mature leaf-preferred expression of *AQP*. Difference of expression level is indicated as Log2 ratio of young relative to mature leaf samples. Differential *AQP* transcript accumulations between young and mature leaves were hierarchically clustered using Euclidean distance. Each row corresponds to an *AQP* gene. Color scale depicts Log2 ratio value: Green represents young leaf-preferred expression and red represents mature leaf-preferred expression. Columns correspond to 10 comparisons from distinct experiments. Samples were collected from *Populus balsamifera* AP1006 trees grown on soil without water limitation (2: midday harvest) or from *P. fremontii x P. angustifolia trees* grown in hydroponics (genotype RM5 wounded (5), RM5 control (9), genotype 3200 grown with 0.125 mM nitrogen (8) or with 5 mM nitrogen (7), genotype 1979 grown with 0.125 mM nitrogen (4) or with 5 mM nitrogen (6)). During a time-course study carried on 30-year old *P. deltoides* tree, foliar tissues were collected in March, prior to the terminal bud opening, in April, May and June 2006. Impact of leaf maturity was assessed at three time points during leaf expansion, namely in March (10), May (3) and April (1) relatively to June (mature leaf). Microarray data were found under the series accession numbers: GSE13990 (2), GSE16786 (4 to 9) and GSE24349 (1, 3 and 10).

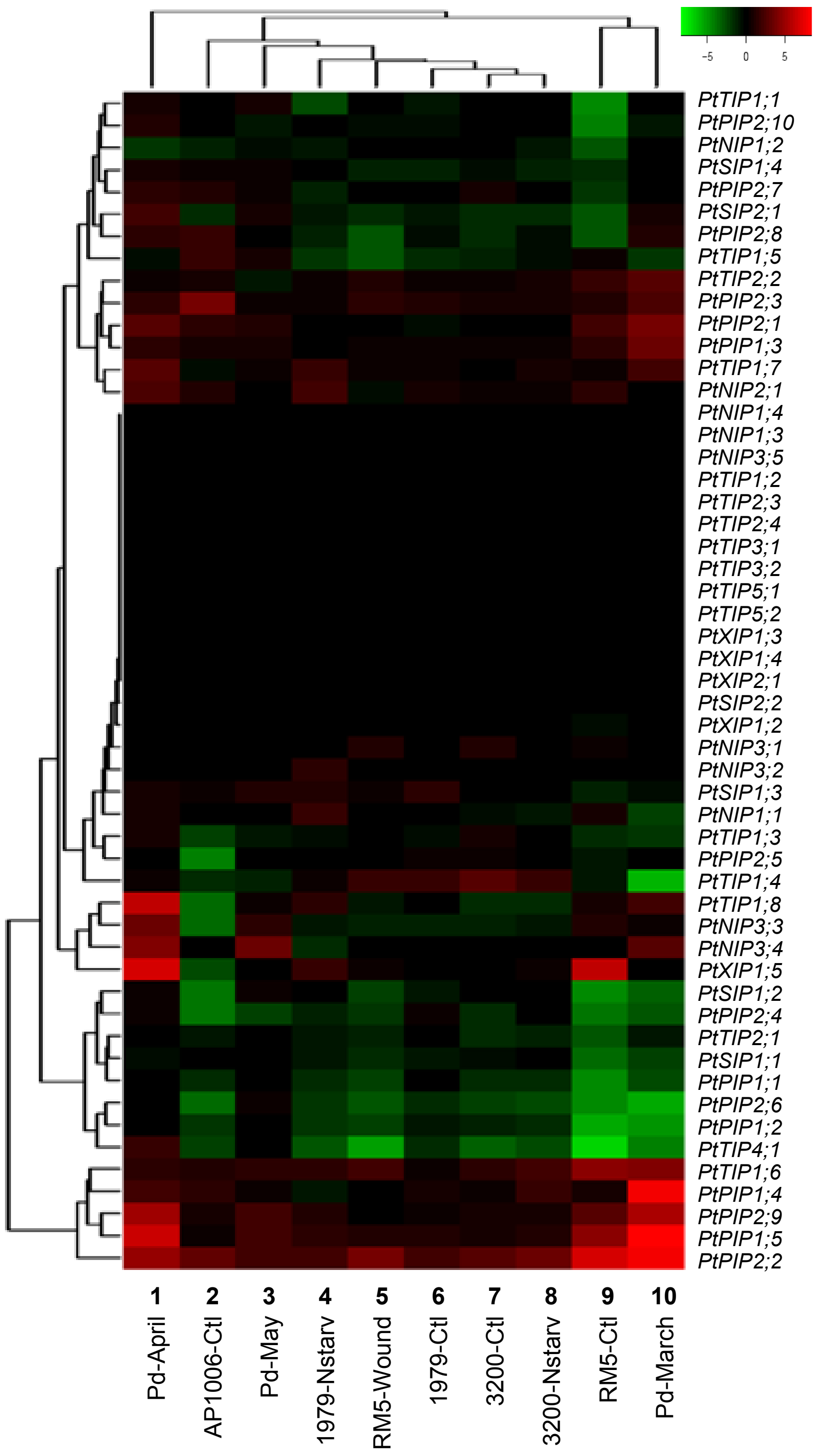

−5
0
5
PtTIP1;1
PtPIP2;10
PtNIP1;2
PtSIP1;4
PtPIP2;7
PtSIP2;1
PtPIP2;8
PtTIP1;5
PtTIP2;2
PtPIP2;3
PtPIP2;1
PtPIP1;3
PtTIP1;7
PtNIP2;1
PtNIP1;4
PtNIP1;3
PtNIP3;5
PtTIP1;2
PtTIP2;3
PtTIP2;4
PtTIP3;1
PtTIP3;2
PtTIP5;1
PtTIP5;2
PtXIP1;3
PtXIP1;4
PtXIP2;1
PtSIP2;2
PtXIP1;2
PtNIP3;1
PtNIP3;2
PtSIP1;3
PtNIP1;1
PtTIP1;3
PtPIP2;5
PtTIP1;4
PtTIP1;8
PtNIP3;3
PtNIP3;4
PtXIP1;5
PtSIP1;2
PtPIP2;4
PtTIP2;1
PtSIP1;1
PtPIP1;1
PtPIP2;6
PtPIP1;2
PtTIP4;1
PtTIP1;6
PtPIP1;4
PtPIP2;9
PtPIP1;5
PtPIP2;2
1 Pd-April
2 AP1006-Ctl
3 Pd-May
4 1979-Nstarv
5 RM5-Wound
6 1979-Ctl
7 3200-Ctl
8 3200-Nstarv
9 RM5-Ctl
10 Pd-March

Figure S3
