# Supplementary material for: Developmental and Environmental Regulation of Aquaporin Gene Expression across Populus Species: Divergence or Redundancy?
Source: PLoS One. 2013 Feb 5;8(2):e55506. doi: 10.1371/journal.pone.0055506 (PMC3564762; doi:10.1371/journal.pone.0055506)
Supplement: Figure S4 — Transcriptional regulation of AQP expression under abiotic challenges. (PDF) [file pone.0055506.s004.pdf]

Figure S4. Transcriptional regulation of *AQP* expression under abiotic challenges. Regulation of expression is indicated as Log2 ratio of treated samples relative to control samples and is visualized as heatmaps. Differential *AQP* transcript accumulations between samples were hierarchically clustered using Euclidean distance. Each row corresponds to an *AQP* gene. Color scale depicts Log2 ratio value: Green represents down-regulation and red represents up-regulation in treated samples relatively to controls. **Heatmap A: *AQP* responses to water deficit and osmotic stress.** Columns correspond to 48 comparisons carried on leaf (L), root (R) or xylem (X). Distinct experimental set-ups of drought and osmotic stress are analysed. Column legends summarized the following information. Poplar hybrids (*Populus deltoides x P. nigra* Soligo or Carpaccio) after 36h drought (EAR) and after 10-day water deficit of mild (LMI) or moderate intensity (LMO) (GSE17223 and GSE17230). Soligo or Carpaccio responses to 10-day moderate soil water deficit (LMI2, GSE17226). Soligo after 3-day of 200g/L PEG osmotic stress (PEG, GSE17225). Drought responses of *P deltoides x P. nigra* DN34 and *P. maximowiczii x P. nigra* NM6 over a diurnal period - leaf being collected at predawn, midday, lateday and midnight (GSE15242). Drought response of poplar hybrids: DN34 (*P deltoides x P. nigra*), Walker (*P. laurifolia x P. nigra*) and Okanese (Walker x (*P. laurifolia x P. nigra*)), with two samples per hybrid coming from distinct geographic locations, analysed at predawn and midday (GSE27693). Drought response of *P. balsamifera* (genotypes AP947, AP1005, AP1006, AP2278, AP2298 and AP2300) at predawn and midday (GSE21171). Drought response of young differentiating xylem in *P. canescens* INRA717 1-B4 (GSE20061). **Heatmap B: *AQP* responses to salt and hypoxia stresses.** Columns correspond to 9 comparisons between treated and respective control samples, carried on leaf (L), root (R) or xylem (X). Salt responses of *P. euphratica* genotype B2 and of *P. canescens* genotype INRA717 1-B4 analysed in xylem after a 2-week exposure to 100 mM NaCl (E-MEXP-2031). Salt response of *P. euphratica* in cob root tips developed under 150 mM NaCl (E-MEXP-2234). Salt response of *P. canescens* INRA717 1-B4 in control or mycorrhized with *Paxillus involutus* (myc) fine roots, both developed under 150 mM NaCl (E-MEXP-1874). Hypoxia response of *P. canescens* (flooded root system) at three time points in roots (65h, 24h, 168h) and at 168h in leaf (GSE13109). **Heatmap C: *AQP* responses to leaf wounding.** Columns correspond to 4 comparisons between treated and respective control samples. Leaf (leaf plastochron index –LPI 8-12) of hydroponically-grown trees of *P. fremontti x P. angustifolia* RM5 were wounded and samples were collected 90h or 1 week afterward. Wounding responses were analysed in root tip (Rtip: apical 1 cm) and in mature leaves (L: LPI 5) after 90h, and in young (YL: LPI 0-1) and mature leaves (L: LPI 5) after 1 week (GSE16786). **Heatmap D: *AQP* responses to starvation and feeding.** Columns correspond to 9 comparisons between treated and respective control samples, carried on mature leaf (L), young leaf (YL) or bark (B). For clarity, samples were not clustered. Starvation responses in *P. fremontti x P. angustifolia genotypes* 1979 and 3200: leaves were collected on trees grown for 4 or 8 weeks on nitrogen deficient medium (Nstarv: 0.125 mM) or on control medium (Ctl: 5 mM nitrogen) (GSE16786). Time-course of bark response to starvation and feeding: defoliated stem of *P. trichocarpa* Nisqually (Nis) were harvested at 0h, 24h, 48h or 72h after the beginning of incubation either in glutamine (Glu: 25 mM), in glucose (Glc: 25 mM), in glutamine and glucose (Glu+Glc 25 mM), or in water (control) (GSE29303). **Heatmap E: *AQP* responses to aluminium stress.** Columns correspond to 3 comparisons between treated and respective control samples and were not clustered. Response to 500 µM aluminium treatment in *P. tremula* (Ptre) root tip collected at three time points (6h, 54h and 256h). Root growth was strongly inhibited at 6 h and partially recovered after 2 days (GSE19297). **Heatmap F: *AQP* responses to embolism and sucrose infiltration.** Response of xylem parenchyma cells from *P. trichocarpa* plants subjected to artificial embolism or sucrose infiltration relatively to control plants (GSE32322).

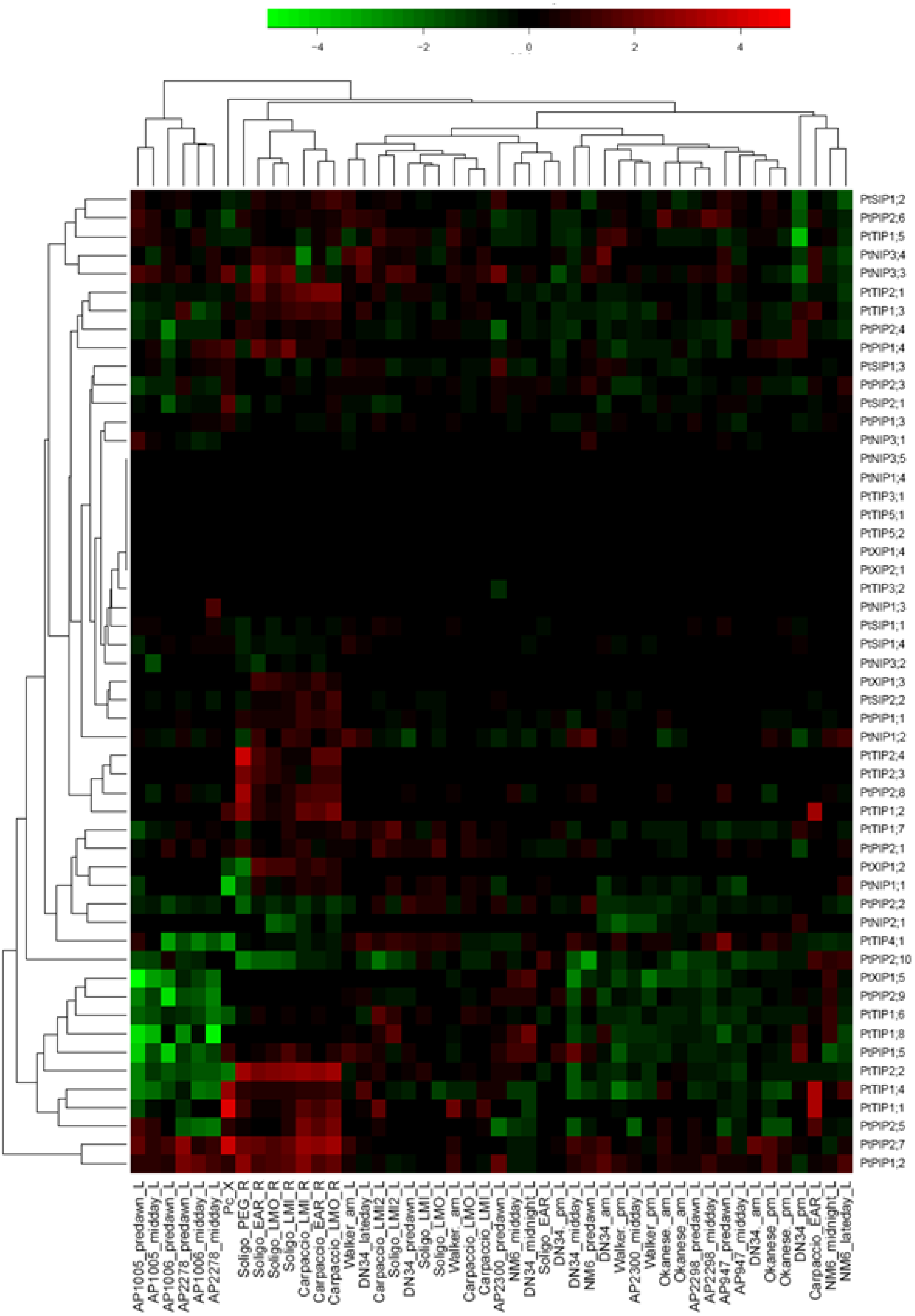

Figure S4A. Water deficit and osmotic stresses

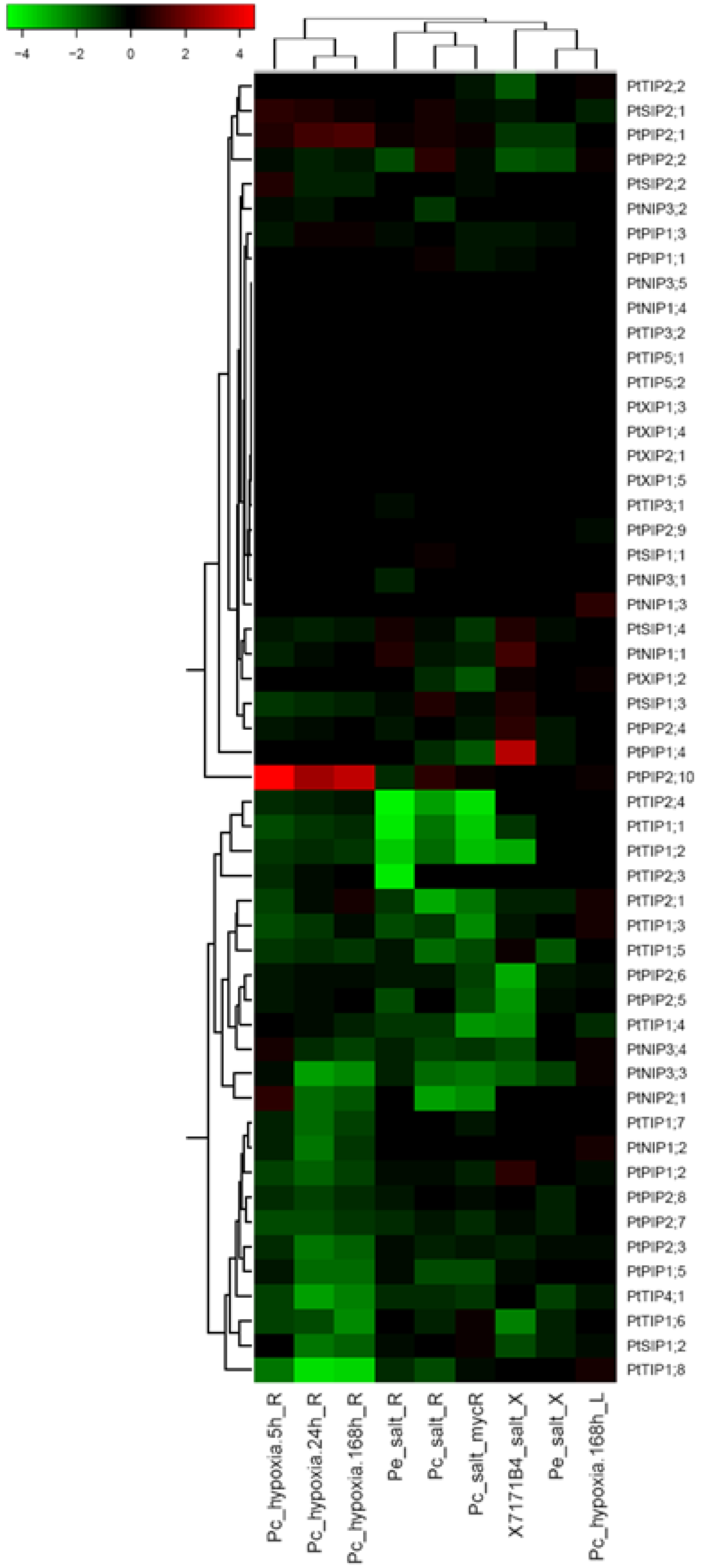

Figure S4B. Salt and hypoxia stresses

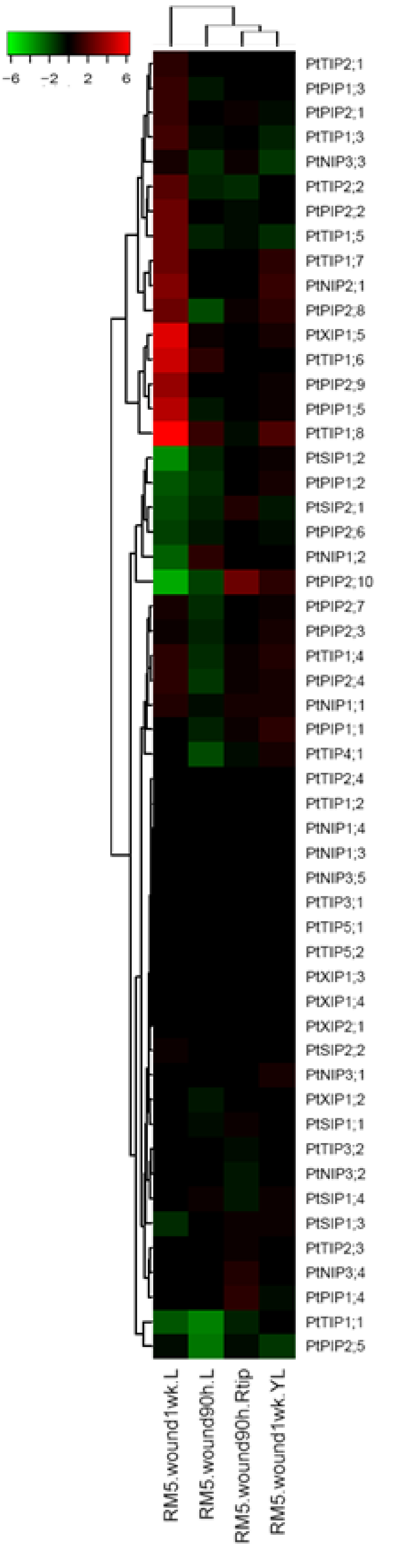

Figure S4C. Leaf-wounding

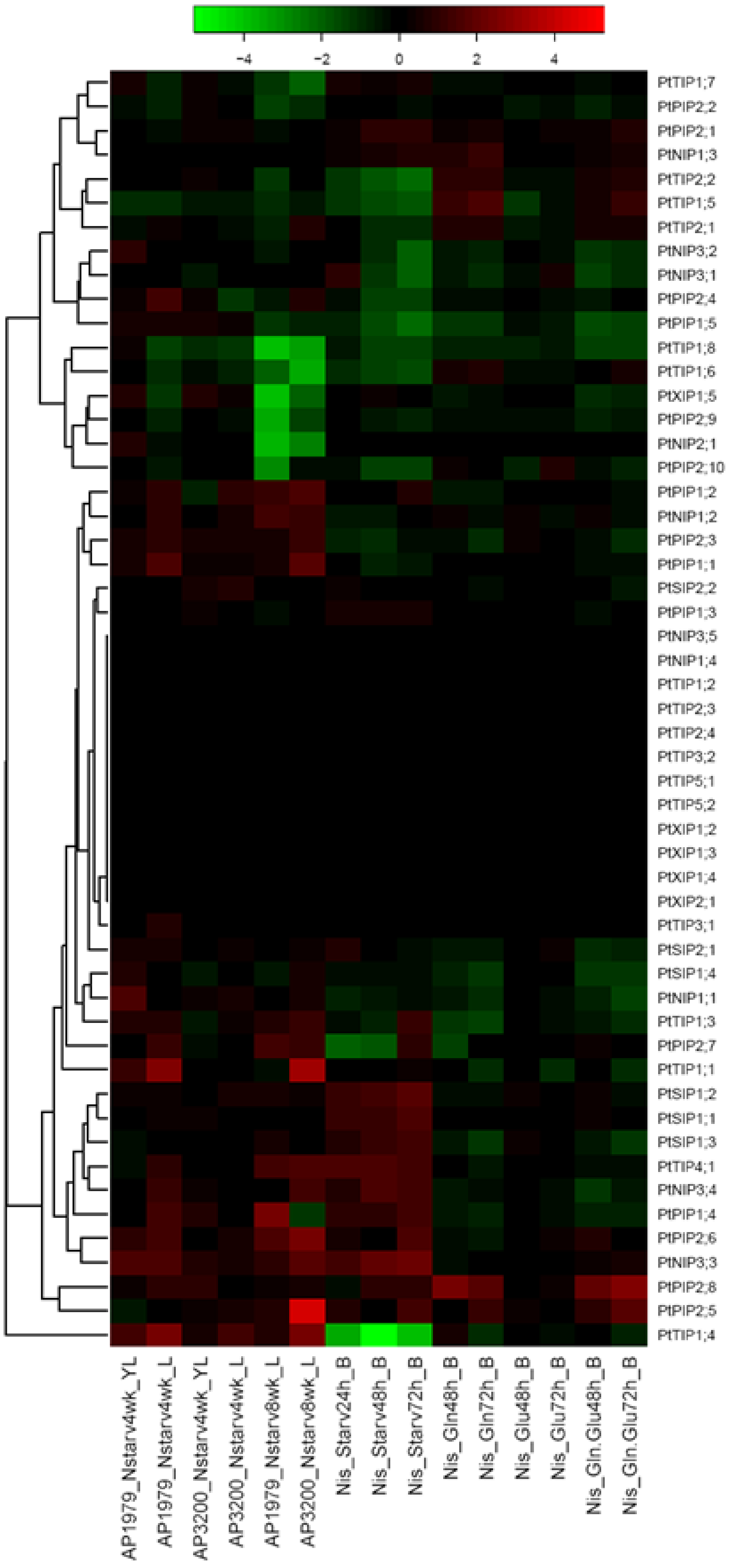

Figure S4D. Starvation & Feeding

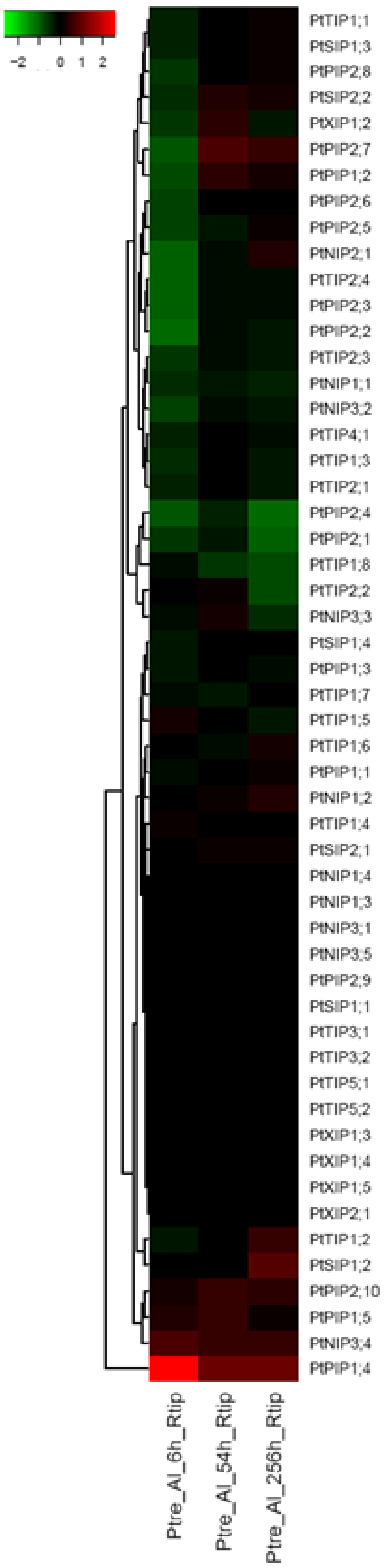

Figure S4E. Aluminium

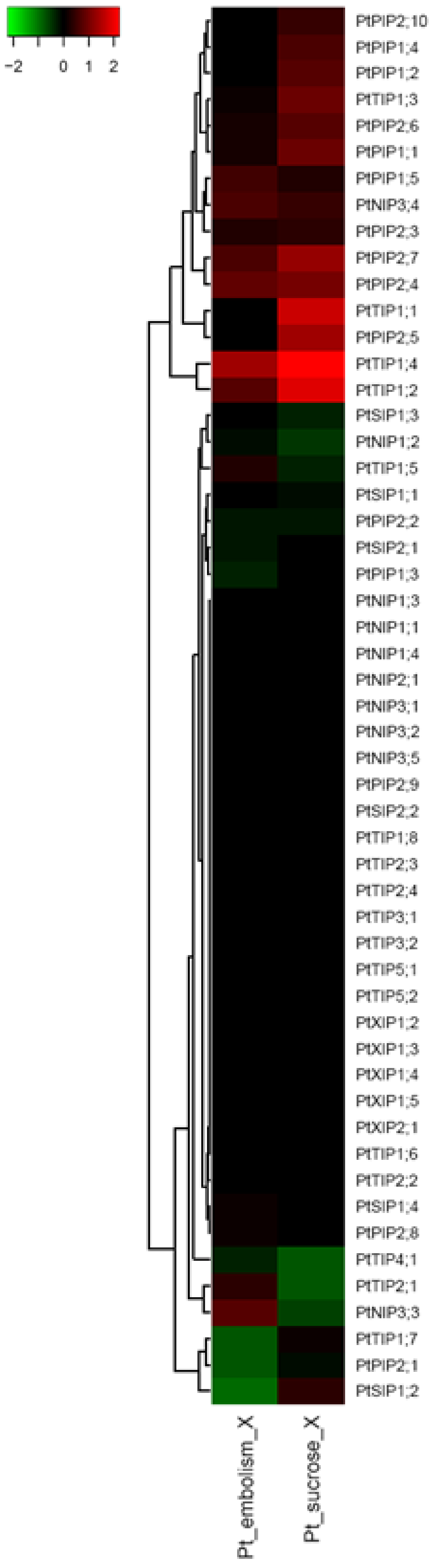

Figure S4F. Embolism and sucrose infiltration
