# Supplementary material for: Developmental and Environmental Regulation of Aquaporin Gene Expression across Populus Species: Divergence or Redundancy?
Source: PLoS One. 2013 Feb 5;8(2):e55506. doi: 10.1371/journal.pone.0055506 (PMC3564762; doi:10.1371/journal.pone.0055506)
Supplement: Figure S5 — Transcriptional regulation of AQP expression in response to hormonal signalling. (PDF) [file pone.0055506.s005.pdf]

Figure S5. Transcriptional regulation of *AQP* expression in response to hormonal signaling. Regulation of expression is indicated as Log2 ratio of treated (or transgenic lines) samples relative to control (or wild type) samples and is visualized as heatmaps. Differential *AQP* transcript accumulations between samples were hierarchically clustered using Euclidean distance. Each row corresponds to an *AQP* gene. Color scale depicts Log2 ratio value: Green represents down-regulation and red represents up-regulation in treated samples relatively to controls. **Heatmap A: *AQP* responses to *in-vitro* manipulation.** Response of stem explant of *P. canescens* INRA717 1-B4 to *in vitro* callus induction after 3 days and 15 days of culture on CIM (callus induction medium, an auxin-rich medium), relatively to stem explant at day 0. Response to *in vitro* shoot induction after 15 days of culture on CIM plus 3 days or 8 days of culture on SIM (shoot induction medium, a cytokinin -rich medium, relatively to 15 days on CIM (GSE12152). **Heatmap B: *AQP* responses to methyl-jasmonate treatment.** *P. tremuloides* cell suspension culture subjected to 48h of methyl jasmonate feeding as compared to control cell suspension culture (GSE16773). **Heatmap C: Regulation of *AQP* expression in *gibberellin*-modified transgenic lines**. Comparison of *AQP* expression in root samples of gibberellin-deficient lines (35S:*PcGA2ox1*) or gibberellin-insensitive lines (35S:*rgl1*) relatively to wild type (*P. canescens* INRA717 1-B4 (GSE16888). As highlighted, the gibberellin-modified lines exhibited contrasting root phenotypes (Gou *et al.,* 2010).

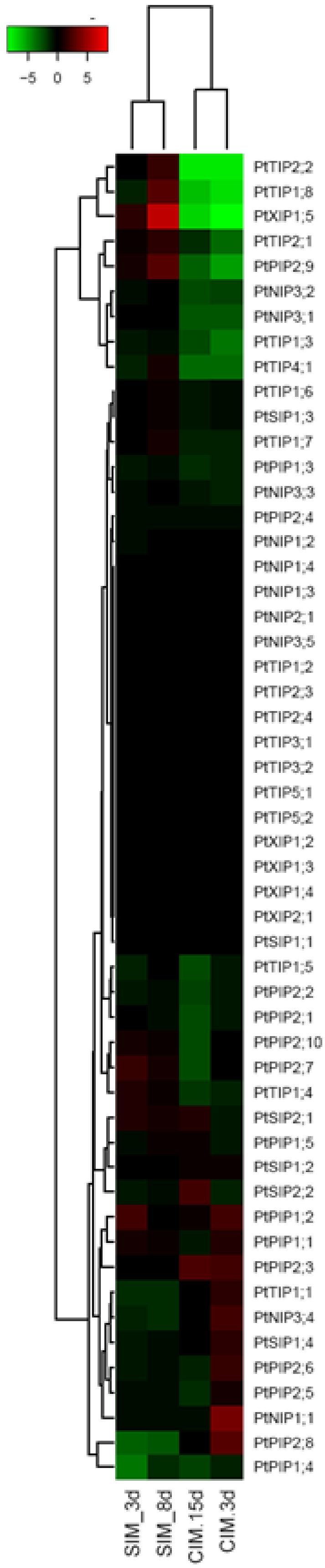

Figure S5A.

***In-vitro* manipulation**

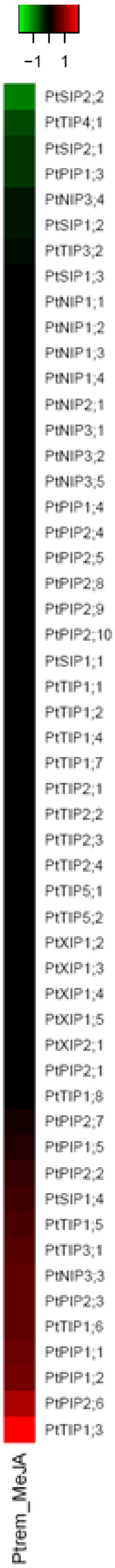

Figure S5B.

Metyl-jasmonate treated

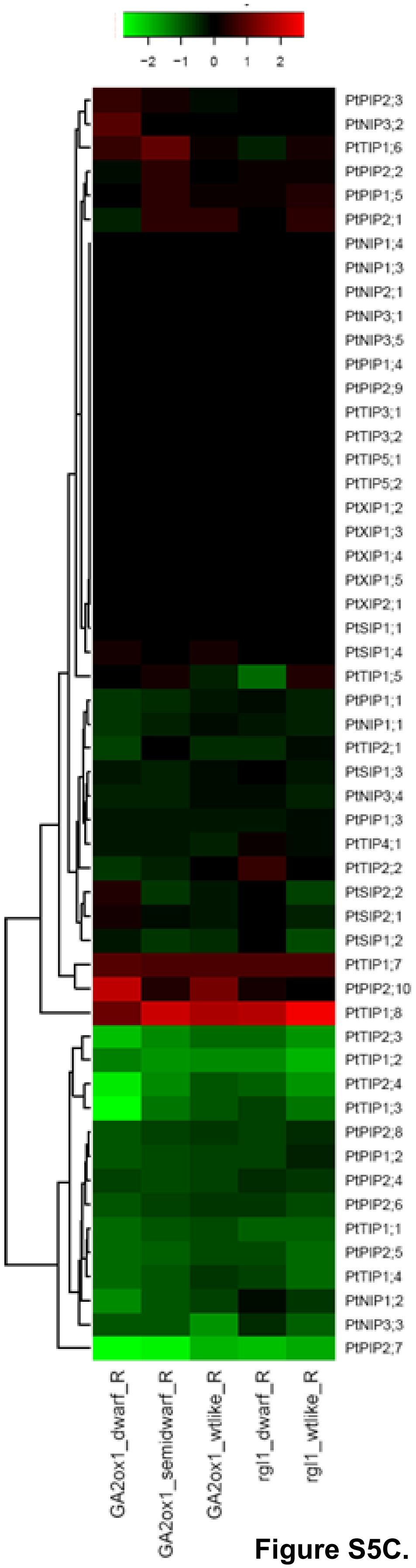

Figure S5C.

***Gibberellin*-modified transgenic lines**
