# Supplementary material for: Developmental and Environmental Regulation of Aquaporin Gene Expression across Populus Species: Divergence or Redundancy?
Source: PLoS One. 2013 Feb 5;8(2):e55506. doi: 10.1371/journal.pone.0055506 (PMC3564762; doi:10.1371/journal.pone.0055506)
Supplement: Figure S6 — Transcriptional regulation of AQP expression under biotic interactions. (PDF) [file pone.0055506.s006.pdf]

Figure S6. Transcriptional regulation of *AQP* expression under biotic interactions. Regulation of expression is indicated as Log2 ratio of treated samples relative to control samples and is visualized as heatmaps. Differential *AQP* transcript accumulations between samples were hierarchically clustered using Euclidean distance. Each row corresponds to an *AQP* gene. Color scale depicts Log2 value: Green represents down-regulation and red represents up-regulation in treated samples relatively to controls. **Heatmap A: *AQP* responses to mycorrhization.** Comparison of mycorrhized *P. canescens* roots (inoculated with *Paxillus involutus*) with non-mycorrhized roots in control conditions and under 150 mM NaCl treatment (E-MEXP-1874). **Heatmap B: *AQP* responses to foliar pathogens.** Columns correspond to 4 comparisons between treated and control leaf discs. Responses of *Populus nigra x maximoviczii NM6 to* infection by biotrophic fungi, *Melampsora larici-populina* (Mlp) or *M. medusae f. sp. Deltoidae* (Mmd), and to a simultaneous infection with both pathogens (GSE9673). Response of *P. canescens* to *M medusae* f.sp. *tremuloidae* (Mmt, GSE16417).

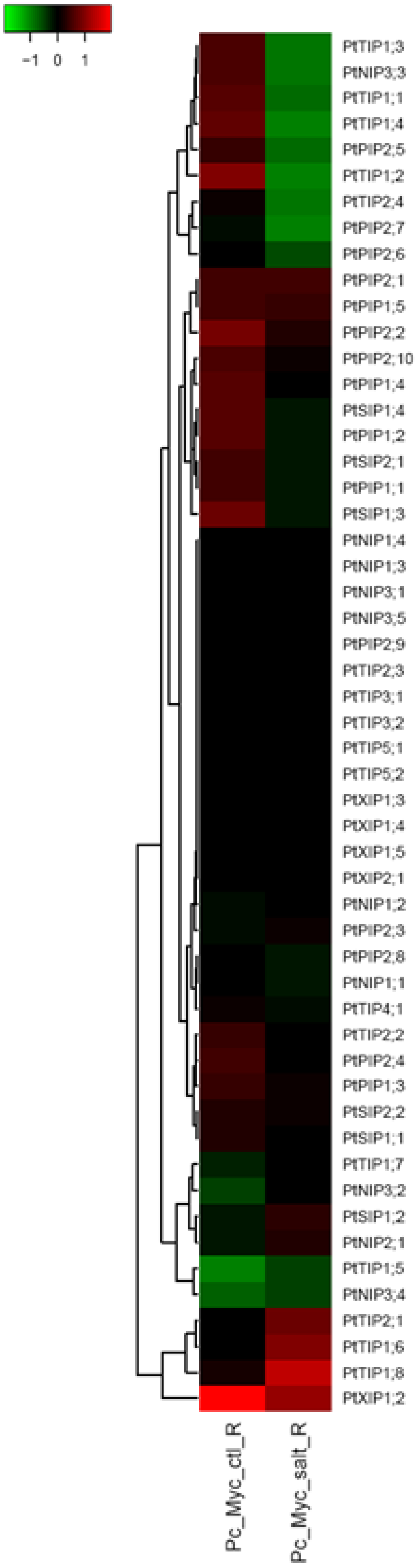

Figure S6A. Mycorrhization

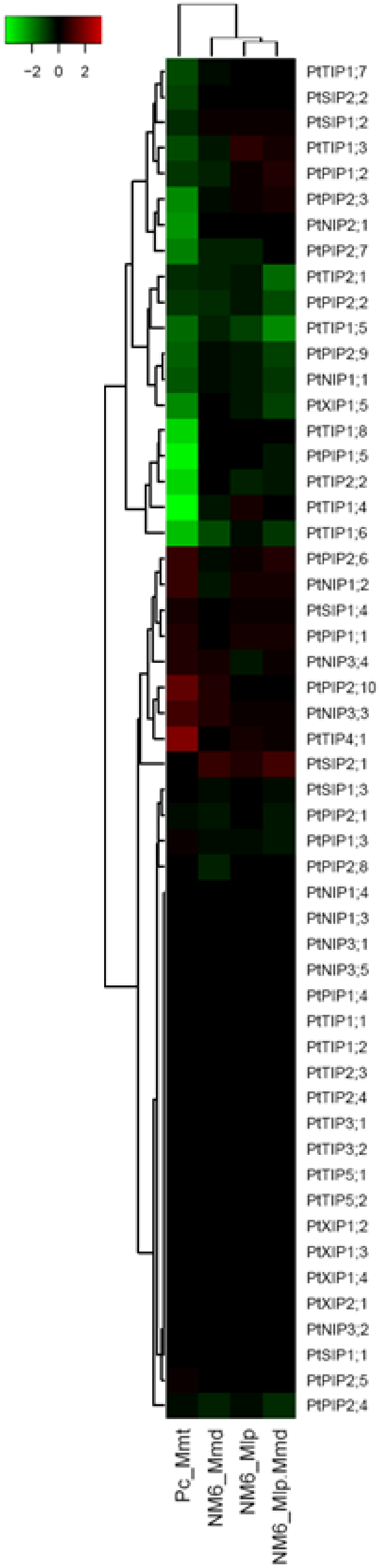

Figure S6B. Foliar pathogens
