# Supplementary material for: Developmental and Environmental Regulation of Aquaporin Gene Expression across Populus Species: Divergence or Redundancy?
Source: PLoS One. 2013 Feb 5;8(2):e55506. doi: 10.1371/journal.pone.0055506 (PMC3564762; doi:10.1371/journal.pone.0055506)
Supplement: Figure S7 — Seasonal or diurnal regulation of AQP expression. (PDF) [file pone.0055506.s007.pdf]

Figure S7. Seasonal or diurnal regulation of *AQP* expression. Relative transcript abundance over time is represented as the Log2 ratio of each time point to the reference time point and is visualized as heatmaps. Differential *AQP* transcript accumulations between samples were hierarchically clustered using Euclidean distance. Each row corresponds to an *AQP* gene. Color scale depicts Log2 value: Green represents lower and red represents higher transcript abundance at each time point relatively to the reference time point. **Heatmap A: seasonal regulation of *AQP* expression.** *Populus deltoides* preformed leaves (LB) collected in December, February and March were compared to samples collected in September (GSE24349). *P. deltoides* catkin buds (FB) collected in May, June, July and August were compared relatively to those collected in April (GSE29335). *P. trichocarpa* stem collected in winter compared to stem collected in summer (GSE21480). **Heatmap B: Diurnal regulation of *AQP* expression.** Columns correspond to 22 comparisons between temporal points from distinct experimental series. Samples were collected from control trees grown in soil without water limitation. Column legends summarized the following information. Diurnal regulation in mature (L) of *P. deltoides x P. nigra* DN34 and *P. maximowiczii x nigra* NM6 (leaves being collected at predawn, midday, lateday and midnight, GSE15242). Midday/predawn contrasts in mature leaves of poplar hybrids DN34 (*P. deltoides x P. nigra*), Walker (*P. laurifolia x P. nigra*) and Okanese (Walker x (*P. laurifolia x P. nigra*)), with two samples per hybrid coming from distinct geographic locations, analysed at predawn and midday (GSE27693). Midday/predawn contrasts in mature leaves of poplar trees of *P. balsamifera* (genotypes, AP947, AP1005, AP1006, AP2278, AP2298 and AP2300, GSE21171).

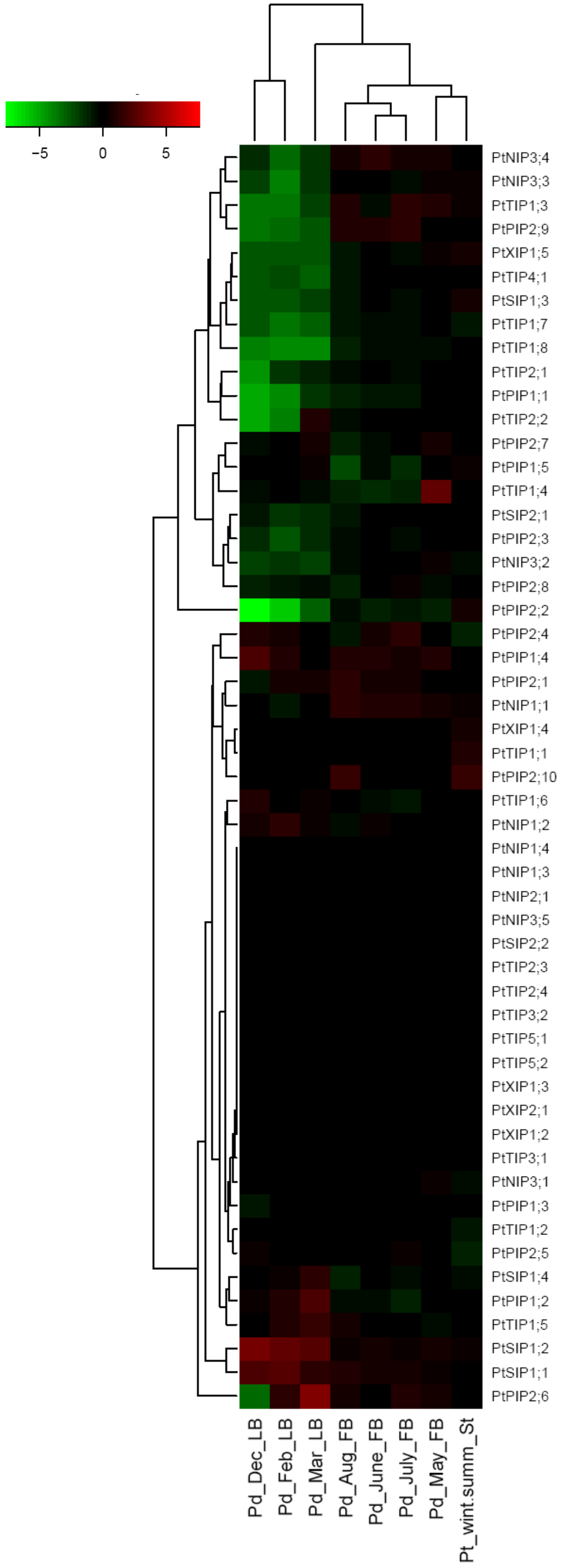

Figure S7A. Seasonal variations

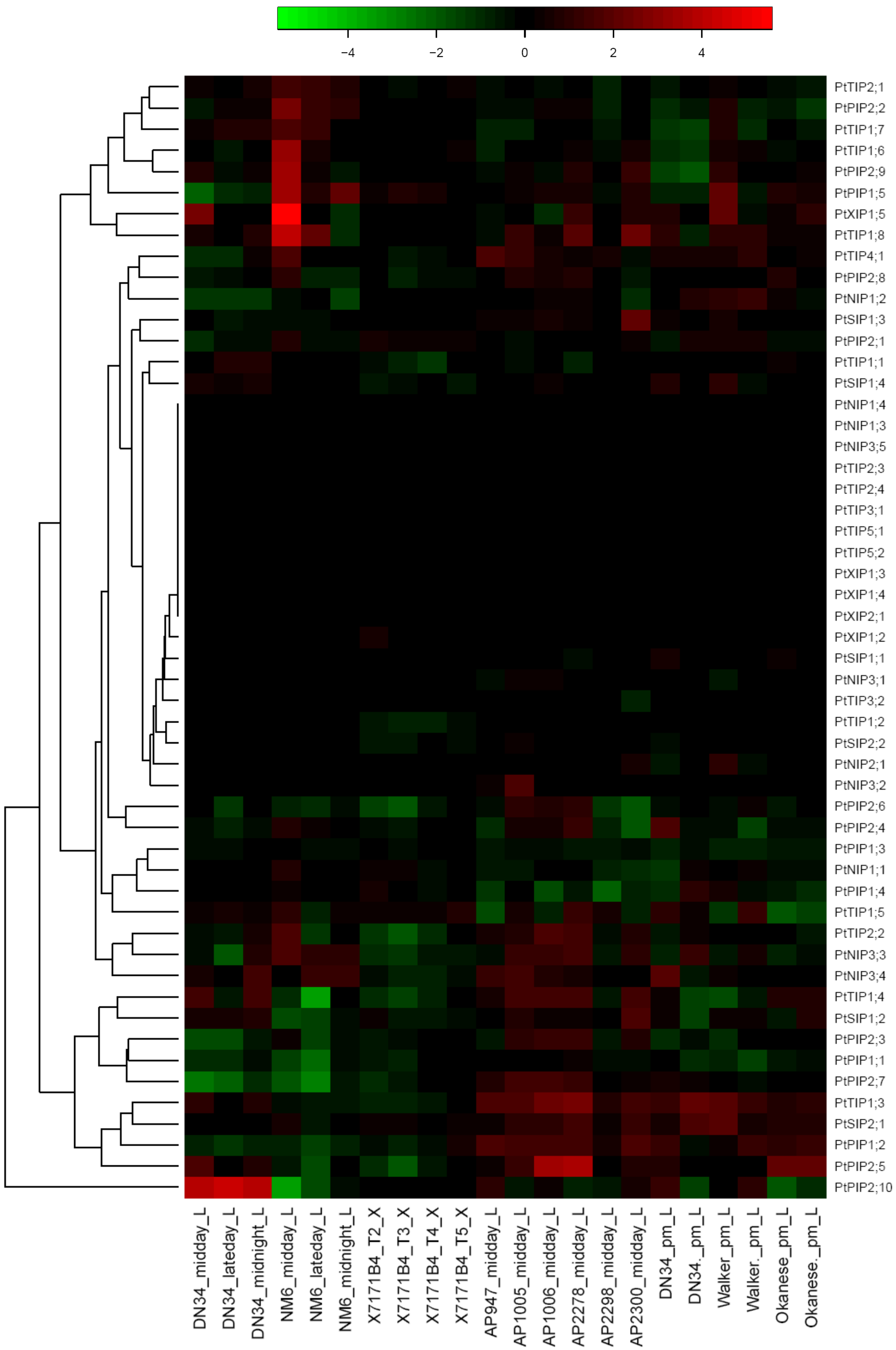

Figure S7B. Diurnal variations
