# Supplementary material for: Developmental and Environmental Regulation of Aquaporin Gene Expression across Populus Species: Divergence or Redundancy?
Source: PLoS One. 2013 Feb 5;8(2):e55506. doi: 10.1371/journal.pone.0055506 (PMC3564762; doi:10.1371/journal.pone.0055506)
Supplement: Figure S8 — Differential AQP expression in response to other cues. (PDF) [file pone.0055506.s008.pdf]

Figure S8. Differential *AQP* expression in response to other cues. Differential expression is indicated as Log2 ratio of treated (or transgenic lines) samples relative to control (or wild type) samples and is visualized as heatmaps. Differential *AQP* transcript accumulations between samples were hierarchically clustered using Euclidean distance. Each row corresponds to an *AQP* gene. **Heatmap A:** Green represents down-regulation and red represents up-regulation in treated samples relatively to control samples. *AQP* expression in Thaxtomin A-habituated resistant cells grown without the toxin for 18 months as compared to control cells. Thaxtomin-A habituated resistant cells displayed a reduced size and growth and had fragmented vacuoles as compared to control cells (GSE17804). **Heatmap B**: Differential expression in defoliated stem (DS) of *ARBORKNOX2*-modified lines (*ARK2: Populus* Class I KNOX homeobox gene), namely *ark2* artificial microRNA transgenic plants and *ARK2* overexpression transgenic plants (GSE17804). **Heatmap C**: Differential expression in leaves of *P. tremula x tremuloides* overexpressing *Flowering Locus T1 or T2,* either following heat promotion of inducible constructs (ProHSP::FT1 and ProHSP::FT2) or constitutive expression constructs *(*P35S::FT1 and P35S::FT2) (GSE24609). **Heatmap D**: Differential expression in leaf discs – inoculated or not with *Melampsora medusae* f. sp. *tremuloidae-* collected from *PtWRKY23*-overexpressing and *PtWRKY23*-underexpressing (RNAi) lines of *P. canescens* (GSE16417). **Heatmap E**: Differential *AQP* expression in several unrelated lines. Differential expression in shoot apex (SA) of Fuzzy mutant (obtained from activation tagged *P. canescens* INRA717 1-B4, GSE21061). The fuzzy phenotype was recapitulated by overexpressing *PtaMYB186* in poplar. Differential expression in leaf of transgenic line expressing 35S:*AtVND7*-*VP16*-GR treated with dexamethazone (GSE25304), in stem tissue (ST) collected from lines overexpressing *Populus* ortholog of *ATHB15/CORONA* or *Populus* ortholog of *REVOLUTA* (GSE19467, an experiment about *Populus* Class III *HDZIPs*), in the shoot apex collected from a line with constitutive overexpression of *EARLY BUD-BREAK1* (P35S::*EBB1*, GSE16495), in young differentiating xylem collected from lines with reduced expression of *Cinnamyl Alcohol Dehydrogenase* (RNAi-*CAD*, GSE27063) and in leaves collected from *CO1*- and *CO2*-overexpressing lines (P35S::*CO1* and P35S::*CO2,* GSE28706).

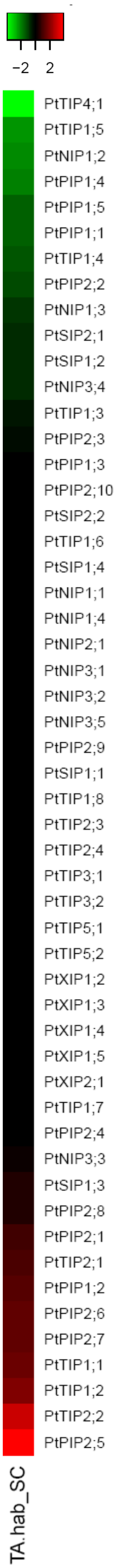

Figure S8A. TA-habituated

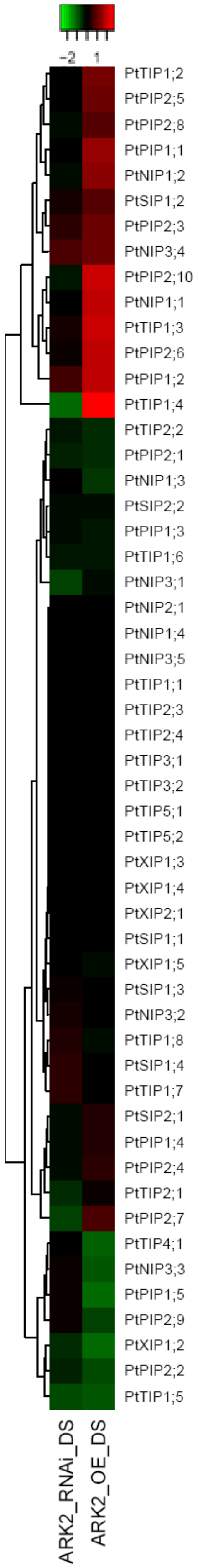

Figure S8 B. ***ARBORKNOX2*-modified**

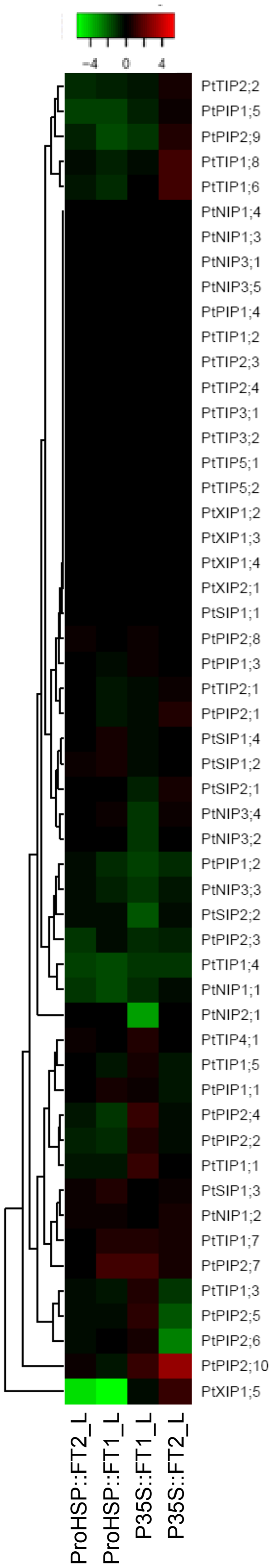

Figure S8C. ***Flowering Locus T*-modified**

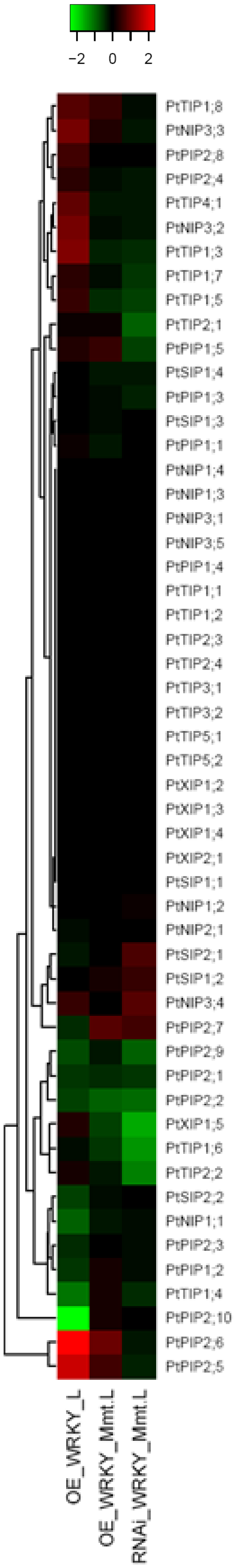

Figure S8D. ***WRKY23*-modified**

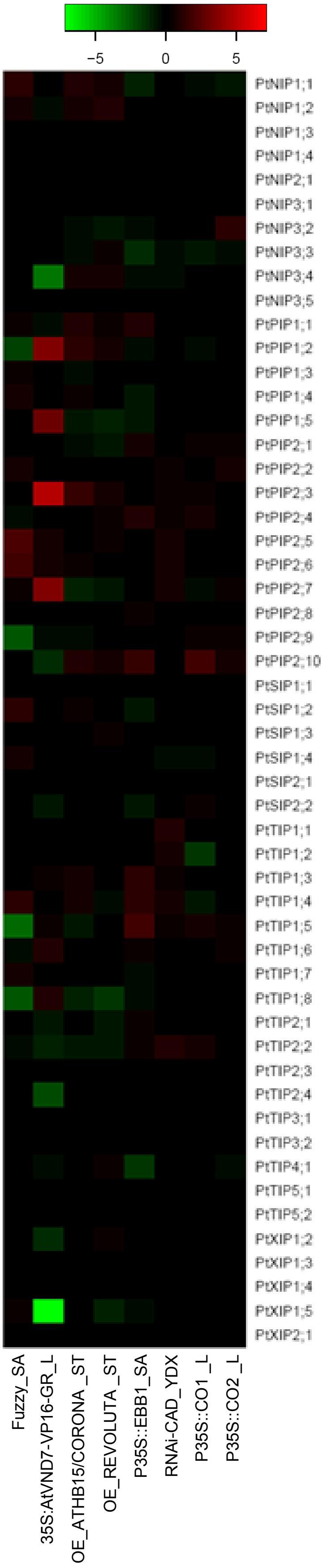

Figure S8E. ***Other transgenic lines***
